# Application of *Pseudomonas mosselii* PR5 Enables Fertilizer Reduction in Rice While Maintaining Yield, Grain Quality, and Soil Nutrient Dynamics

**DOI:** 10.1002/pei3.70182

**Published:** 2026-07-09

**Authors:** Sourav Biswas Shuvo, Razia Sultana, Shah Mohammad Naimul Islam, Imam Mehedi, Md. Azmain Farek Milon

**Affiliations:** ^1^ Department of Agricultural Chemistry Bangladesh Agricultural University Mymensingh Bangladesh; ^2^ Institute of Biotechnology and Genetic Engineering Gazipur Agricultural University Gazipur Bangladesh

**Keywords:** benefit–cost ratio (BCR), grain yield, growth promotion, inorganic fertilizer, nutrients accumulation, plant growth‐promoting rhizobacteria (PGPR), soil fertility

## Abstract

The use of inorganic fertilizers in rice farming has led to increased production costs, reduced nutritional value, and it results in major ecological damage. Harnessing plant growth‐promoting rhizobacteria (PGPR) offers a promising eco‐friendly alternative to conventional fertilization strategies. This study evaluates the potential of *Pseudomonas mosselii* PR5 as a bioinoculant to enhance rice growth under reduced fertilizer inputs in a pot experiment. Two application methods, seed priming (B1) and seed priming combined with bacterial culture filtrate (BCF) foliar spray (B2), were evaluated across four fertilizer regimes: 0% (F0), 50% (F1), 75% (F2), and 100% (F3) of the recommended dose of fertilizer (RDF). Application of PR5 significantly enhanced rice growth, yield, and yield‐related traits across all fertilizer levels and application methods. At 100% RDF, the combination of seed priming with foliar application of PR5 resulted in marked increases in plant height (4.3%), biomass (64.4%), chlorophyll content (47.4%), grain yield (48%), and benefit–cost ratio (67.6%) compared to the 100% RDF alone. Notably, the combined PR5 application at 75% RDF showed similar agronomic performance and yield as in the 100% RDF treatment, indicating 25% less fertilizer requirement for maintaining crop profitability. Multivariate analysis also confirmed a strong association of grain and soil nutrients with the combined application of PR5 at 75% and 100% RDF. PR5 application also boosted both grain nutrient content and post‐harvest soil nutrient levels. Thus, this research showed that PR5 application increased crop productivity while improving grain nutritional quality and lowering the need for chemical fertilizers towards sustainable agriculture.

## Graphical abstract

Overuse of agrochemicals negatively affects the health of soil and biodiversity and the quality of water. *Pseudomonas mosselii* PR5 seed priming and foliar application increase nutrient assimilation and pathogen suppression. PR5, when applied with reduced fertilizer rates, enhances rice growth, yield, grain protein, soil organic matter, economic returns, and ecological sustainability.

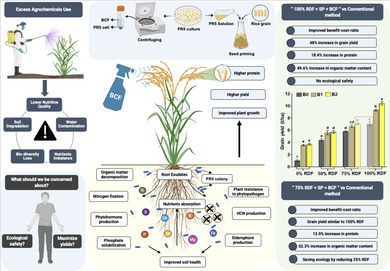

## 1 Introduction

To ensure increasing global food demand, farmers rely on chemical fertilizers and pesticides (Sneha et al. 2018). However, excessive or continuous application of these agrochemicals adversely affects environmental and soil health, leading to reduced fertility, lower organic matter content, diminished beneficial microbial activity, altered soil pH, and increased pest and disease incidence, which reduce crop nutritional quality and edibility (Farnia and Hasanpoor 2015; Tilman et al. 2001). Most of the chemicals are only partially assimilated by the plants, resulting in their continuous accumulation in the soil. Chemical fertilizers typically contain nitrate, phosphate, potassium, ammonium salts, heavy metals (Cu, Cd, Hg, Ni), and radionuclides, most of which are only partially assimilated by plants, resulting in accumulation in the soil (Savci 2012). These residual chemicals contribute to eutrophication, groundwater contamination, and greenhouse gas emissions, including NH_3_ and N_2_O (Ajmal et al. 2018). Plants often utilize < 50% of applied nitrogen, with the remainder increasing susceptibility to rice blast, grain chalkiness, and poor cooking quality (Lassaletta et al. 2014; Long et al. 2000; Zhang et al. 2022). In addition, excessive use of chemical fertilizer pollutes water bodies and causes substantial human health risks, including cancer, reproductive abnormalities, and hormonal disruption (Lassaletta et al. 2014; Hossain et al. 2022).

A promising approach to achieving high crop yield is the use of plant growth‐promoting rhizobacteria (PGPR) as biofertilizers instead of chemical fertilizers. These microbes enhance nutrient availability, increase root biomass and surface area, and improve nutrient uptake through biological nitrogen fixation, phosphate solubilization, and phytohormone production, including IAA, cytokinins, and gibberellins (Malusá et al. 2012; Sultana et al. 2024a; Sultana et al. 2024b). PGPR can also enhance plant tolerance to abiotic stresses such as salinity (Shultana et al. 2022) and drought (Chieb and Gachomo 2023), and protect against diseases via mechanisms like phytohormone production, ACC deaminase activity, antioxidant enzymes, siderophores, exopolysaccharide secretion, and heavy metal immobilization (Kaushal and Wani 2016). By stimulating these biological activities, PGPR improve soil microbial diversity and function (Grover et al. 2011; Milošević et al. 2012; Rong et al. 2025), enhance plant health in saline soils, and support disease control (Lugtenberg et al. 2013; Pliego et al. 2011). These traits make PGPR effective biofertilizers that reduce agrochemical dependence and promote nutrient‐rich crop production (Farnia and Hasanpoor 2015).

*Pseudomonas putida* is one of the more promising genera among the well reported PGPR (Costa‐Gutierrez et al. 2022). The bacteria of these groups are found to be able to promote plant growth as well as effective against biotic and abiotic stresses such as drought and salinity (Egamberdieva and Kucharova 2009; Sandhya et al. 2010). The current research employed a bacterial strain *Pseudomonas mosselii* (PR5), an endophytic bacterium in the *Pseudomonas putida* group, because of its excellent plant growth‐promoting (PGP) qualities. In a laboratory study, it showed several PGP characteristics, including high synthesis of indole‐3‐acetic acid (IAA), nitrogen fixation, siderophore production, HCN production, and solubilization of phosphate and zinc (Sultana et al. 2023). Later, another investigation showed that the isolate boosts rice growth, development, and yield under field conditions through seedling priming, root drenching, and cell‐free culture filtrate foliar application (Sultana et al. 2024c). However, it remains unclear whether the PR5 strain exhibits a remarkable response when applied alongside reduced doses of chemical fertilizers. This uncertainty raises two critical questions: (1) Can PR5 be effectively utilized under sub‐optimal fertilizer conditions to counter the prevailing trend of excessive chemical fertilizer use? (2) Is it possible to harness the bacterium's potential benefits in nutrient uptake and grain quality enhancement when combined with lower fertilizer inputs? Accordingly, the hypothesis was that application of PR5 will lower the amount of chemical fertilizer usage and the overall cost of production. The purpose of this study was to determine whether reduced doses of inorganic fertilizer, when combined with the microbial inoculant PR5, could achieve optimal plant growth, yield, and nutrient uptake comparable to full fertilizer rates, and to identify the minimum level of chemical fertilizer that can be applied alongside PR5 without compromising yield and maintaining soil health.

## 2 Materials and Method

### 2.1 Bacterium and Preparation of Bacterial Inoculant

An indigenous bacterium, *Pseudomonas mosselii* PR5 (NCBI accession number MZ540030), was isolated in our previous experiment (Sultana et al. 2023) and was found to be capable of producing IAA, fixing N_2_, producing siderophore, and solubilizing phosphate, zinc, and silicon (Sultana et al. 2024c). For bio‐inoculation, a freshly prepared bacterial solution containing 10^8^–10^9^ CFU/mL was employed.

### 2.2 Soil Preparation and Planting Material

Soil was collected from a nearby agricultural field at 0 to 15 cm depth. After removing undesirable substances, the physicochemical characteristics of the initial soil sample were determined. The pH and EC were determined by the method of Jackson (1973). The amount of organic carbon in the soil was measured by the process developed by Page (1982). To calculate the organic matter content, the organic carbon was multiplied by the van Bemmelen factor 1.73. Total N in the soil was determined using the Semi‐micro Kjeldahl technique (Bremner 1965). The Olsen technique was used to extract the available P from the initial soil (Olsen 1954). Available S was extracted using CaCl_2_ (Chesnin and Yien 1951). Ammonium acetate solution (1 N NH_4_OAc) at pH 7 was used to extract exchangeable Ca, Mg, and K, according to Black et al. (1965). The DTPA extraction method was employed to determine the available Fe, Zn, and Mn (Lindsay and Norvell 1978). The physiochemical attributes of the initial soil were listed in Table S1.

### 2.3 Experimental Procedure

The rice variety BRRI dhan‐29 was used in a pot experiment. The experiment was conducted using pots measuring 18 cm in depth, 15 cm in diameter at the top, and 13 cm in diameter at the bottom. Each pot held seven kilograms of soil that had been applied with urea, TSP, MOP, and gypsum in the amounts suggested for HYV rice (var. BRRI dhan‐29) in accordance with the Fertilizer Recommendation Guide of Bangladesh (Ahmmed et al. 2018). Here, three different reduced rates of fertilizer doses were used along with the full rate of the recommended dose of fertilizer (RDF), which were as follows: F0 = 0% RDF, F1 = 50% RDF, F2 = 75% RDF, and F3 = 100% RDF. In each fertilizer dose, there were two treatments of bacterial inoculation; one treatment was B1 = bacterial seed priming (SP), another was B2 = seed priming and bacterial culture filtrate (BCF) foliar application (SP + BCF), and the third was the uninoculated control. Therefore, in total there were 12 treatments, namely, F0B0, F0B1, F0B2, F1B0, F2B1, F2B2, F3B0, F3B1, F3B2, F4B0, F4B1, and F4B2. The detail of the treatment combinations and their corresponding code used in the experiment is given in Table 1.

**TABLE 1:** Details of the treatment combinations and their corresponding code used in the experiment.

Treatments code	Fertilizer doses	Bacterial application
F0B0	0%	Uninoculated control (No Bacteria)
F0B1	0%	Seed Priming (SP)
F0B2	0%	Seed Priming (SP) + Bacterial Culture filtrate (BCF) foliar applications
F1B0	50%	Uninoculated control (No Bacteria)
F1B1	50%	Seed Priming (SP)
F1B2	50%	Seed Priming (SP) + Bacterial Culture filtrate (BCF) foliar applications
F2B0	75%	Uninoculated control (No Bacteria)
F2B1	75%	Seed Priming (SP)
F2B2	75%	Seed Priming (SP) + Bacterial Culture filtrate (BCF) foliar applications
F3B0	100%	Uninoculated control (No Bacteria)
F3B1	100%	Seed Priming (SP)
F3B2	100%	Seed Priming (SP) + Bacterial Culture filtrate (BCF) foliar applications

Each treatment was replicated thrice, totaling 36 pots. The experiment was conducted following a completely randomized design (CRD) in a net house. The Table S2 provides daily weather information from the closest meteorological station, including temperature and relative humidity, during the entire period of the experiment. For seed priming, a batch of seeds was soaked in PR5 bacterial solution for roughly 12 h, while another set was soaked in warm distilled water for control. Both batches of soaked seeds were then placed on different petri dishes to germinate properly. After a few days, the perfectly germinated seedlings from both groups were sown into soil within two distinct trays and allowed to grow seedlings. 30‐day‐old seedlings were transplanted to their appropriate pots according to the treatments. After 25 and 50 days of transplanting, bacterial culture filtrate (BCF) was applied in the SP + BCF treatments. To prepare the bacterial culture filtrate (BCF) solution, at first, bacterial liquid culture of approx. 10^8^–10^9^ CFU mL^−1^ was centrifuged. Afterwards, the supernatant was collected, filtered, and blended with 0.5% carboxymethyl cellulose (CMC). The prepared solution was then sprayed over the rice plants at 50 mL/plant at the active tillering stage (25 DAT) and at the flower initiation stage (50 DAT), ensuring full coverage of the leaves by the spray droplets with a high‐density plastic hand sprayer of adjustable nozzle type from mist to stream (Model: Garden Hand Sprayer 2 L).

The intercultural operations were carried out when necessary. Agronomical data including plant height, number of tillers, and number of panicles were recorded prior to harvesting. The harvesting was done on the 119th day after transplanting. The shoot, root, and grain parts were then separated carefully and the fresh weights were taken. The flag leaf and root samples were oven‐dried at 70°C for 48 h, and the dry weight of the samples was recorded.

### 2.4 Determination of Chlorophyll Contents

In order to estimate the amount of chlorophyll in the plants at the heading stage, fresh leaf samples were collected. Spectrophotometric analysis was used to determine the chlorophyll a, chlorophyll b, and total chlorophyll in the samples (Arnon 1949; Lichtenthaler and Wellburn 1983). Relative chlorophyll content (SPAD index) was measured using a SPAD chlorophyll meter (Model: FT Green LLC, Wilmington, DE, USA) following the technique as outlined by Yadava (1986).

### 2.5 Grain Proximate Analysis

The Association of Official Analytical Chemists (AOAC 2004) procedures were used to determine the grain moisture, ash, and fat contents. Total carbohydrates were determined by the method developed by the Food and Agriculture Organization (FAO 2004). The protein content was calculated from the amount of nitrogen in grain by multiplying by the factor of 6.25; the nitrogen content was previously determined using the semi‐micro Kjeldahl method (Bremner 1965).

### 2.6 Extraction of Plant and Post‐Harvest Soil Sample

The dry and ground samples were extracted following the wet oxidation process using a mixture of HNO_3_ and HClO_4_ (Jackson 1973). For total *N*, the semi‐micro Kjeldahl technique was used to extract the samples as described earlier. The post‐harvest soil samples were prepared for extraction and soil‐available nutrient determination following the same protocols as in the initial soil sample.

### 2.7 Nutrients Analysis

The amount of nitrogen in plant and soil extract was determined by the semi‐microkjeldahl method (Bremner 1965). The phosphorus concentration was ascertained by spectrophotometric method (Page 1982). The amount of potassium was determined by a flame emission spectrophotometer according to Ghosh et al. (1983). The sulfur content was evaluated by the turbidimetric method (Tandon 1993). The Ca and Mg contents were determined by the complexometric titration method (Page 1982). The concentrations of iron (Fe) and zinc (Zn) were measured by an atomic absorption spectrophotometer (model: Shimadzu AA700) with a recovery rate of 0.2 ppb at 248.3 nm and 213.9 nm, respectively.

### 2.8 Economic Analysis

The production cost was analyzed to find out the most economic and profitable rate for rice (cv. BRRI dhan29) production. So as to calculate the profitability of the cultivation of rice, the equation that was suggested by Dillon and Hardaker (1980) was used.

$$\eta =\left(Q_{p}+S_{q}\right)–\mathrm{TC}=\left(Q_{p}+S_{q}\right)–\left(\mathrm{TVC}+\mathrm{TFC}\right)$$

where η = Net profit from rice production (Tk/ha), Q = Amount of rice produced (kg/ha), p = Average price of rice (Tk./kg), S = Amount of straw (kg/ha), q = Average price of straw (Tk./kg), TC = Total cost of production (Tk./ha), TVC = Total variable cost (Tk./ha), TFC = Total fixed cost (Tk./ha).

Moreover, the benefit cost ratio (BCR) was calculated using the formula of Tarafder et al. (2020).

$$\mathrm{BCR}=\text{Gross return}\;\left(\mathrm{Tk}/\mathrm{ha}\right)/\text{Total cost of production}\;\left(\mathrm{Tk}/\mathrm{ha}\right)$$

All cost and returns were calculated using the current market prices in BDT with an average exchange rate of 1 USD = 115.44 BDT from January–December 2024 (Source: Bangladesh Bank, 2024).

### 2.9 Statistical Analysis

An analysis of the recorded data was conducted using the statistical program “Minitab 17”. The findings were presented as the standard error (±) and the mean of three replicates. Additionally, to assess the significant differences between the treatments, a two‐way analysis of variance (ANOVA) was conducted. Post hoc analysis was done using Tukey's test (*p* < 0.05). All detailed statistical outputs, including *F*‐values and corresponding *p*‐values, have been provided in a comprehensive Table S3. Visualization of the data was done by the statistical program “GraphPad Prism 8”. Principal component analysis and hierarchical clustering were carried out using the statistical program “R”, version 4.3.0.

## 3 Results

### 3.1 Effect of Bacterial Application on Growth of Rice Under Nutrients Deficient Condition

**FIGURE 1:**
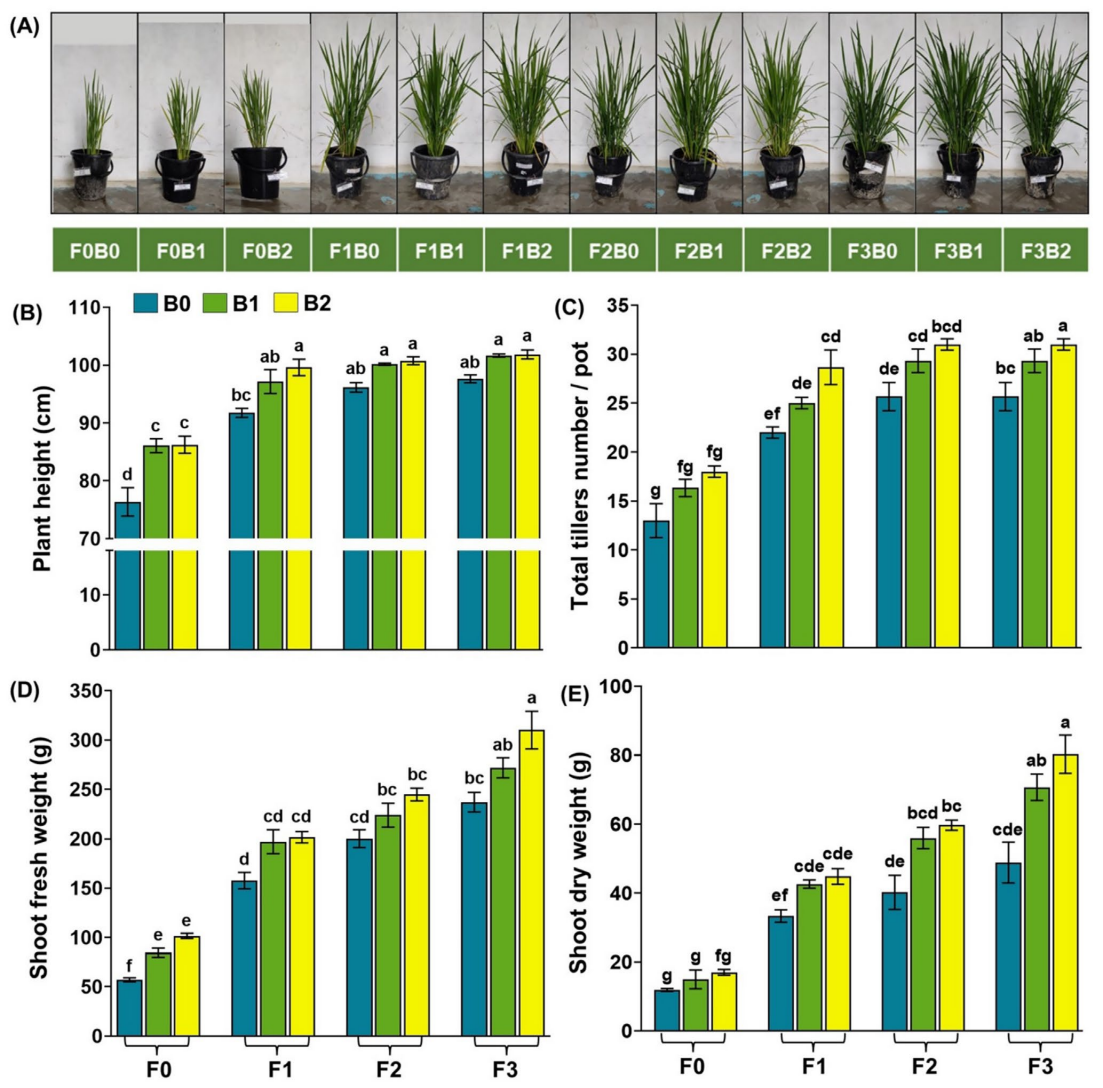
Effect of *Pseudomonas mosselii* PR5 on growth and shoot biomass of BRRI dhan29. (A) Photographic view of rice growth at tillering stage at different treatments, (B) plant height, (C) number of tillers per pot, (D) fresh weight of shoot and (E) dry weight of shoot. Bars (mean ± standard error, *n* = 3) with similar letters are not significantly different according to Tukey's test (*p* < 0.05). B0 = uninoculated control; B1 = seed priming (SP); B2 = seed priming + bacterial culture filtrate foliar application (SP + BCF) and F0 = 0% RDF (recommended dose of fertilizer); F1 = 50% RDF; F2 = 75% RDF; F3 = 100% RDF.

Application of PR5 significantly improved plant height, tiller number, and shoot biomass across all fertilizer levels (Figure 1A). Figure 1A represented the photographic view of plant growth at the tillering stage. Seed priming showed strong growth promotion, further enhanced by BCF foliar application. The highest growth was recorded in F3B2 (100% RDF + PR5), with plant height, tiller number, and shoot dry biomass increased by 4.3%, 21.4%, and 64.3% over the control (F3B0) (Figure 1B–E). Although growth declined with reduced fertilizer (F3B0 > F2B0 > F1B0 > F0B0), PR5 inoculation mitigated these reductions. Notably, F2B2 (75% RDF + PR5) exceeded the conventional 100% RDF control, with 3.2% taller plants and 22.1% higher shoot biomass. Even without fertilizer (F0B2), PR5 enhanced plant height (12.9%), tiller number (38.4%), and shoot biomass (43.3%) compared to F0B0, underscoring its strong growth‐promoting ability under nutrient‐deficient conditions.

### 3.2 Effect of Bacterial Application on Photosynthetic Pigments of Rice Under Nutrients Deficient Condition

**FIGURE 2:**
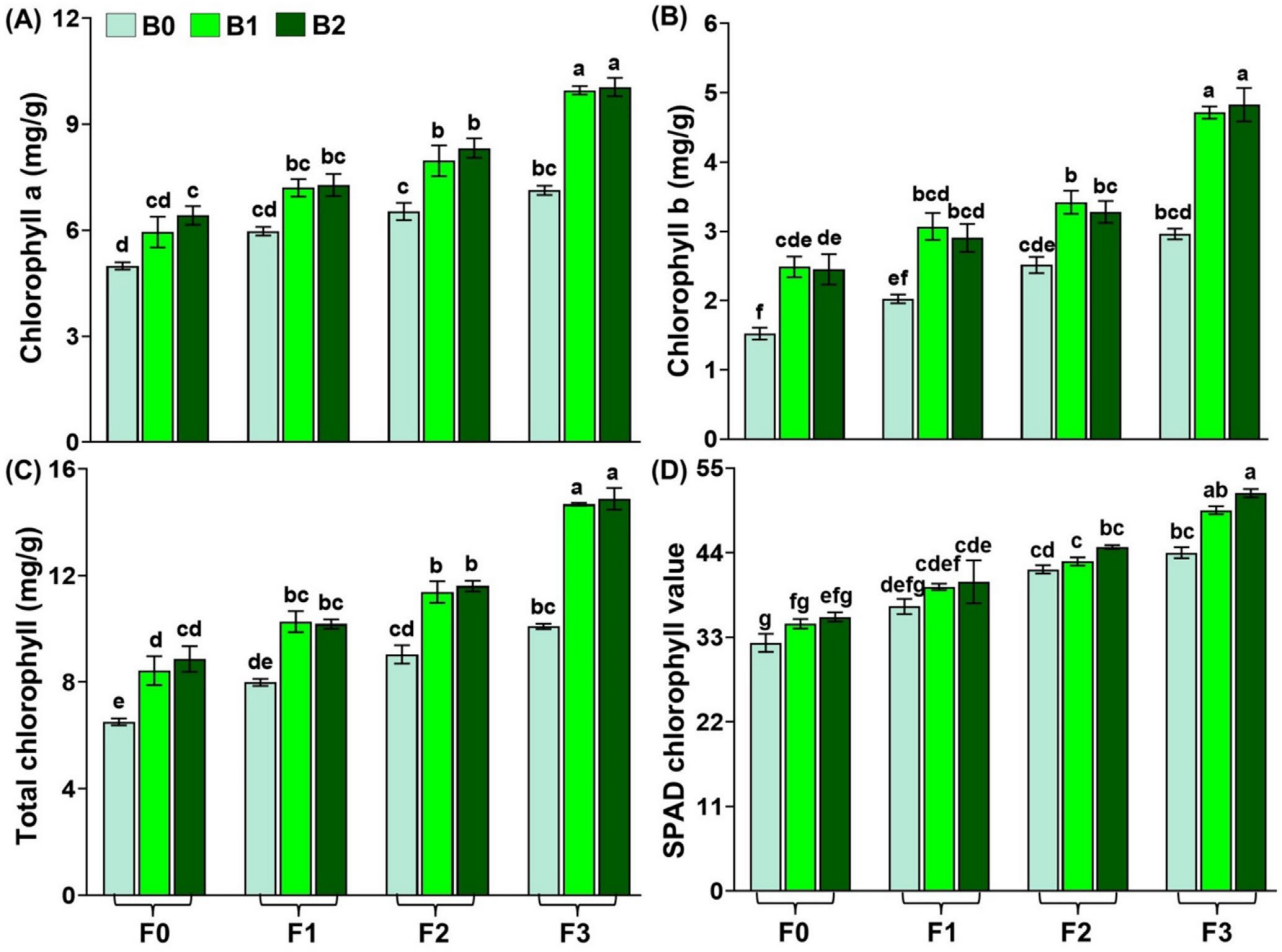
Effect of *Pseudomonas mosselii* PR5 on photosynthetic pigments of BRRI dhan29. (A) chlorophyll a, (B) chlorophyll b, (C) total chlorophyll, and (D) SPAD chlorophyll. Bars (mean ± standard error, *n* = 3) with similar letter are not significantly differed according to Tukey's test (*p* < 0.05). B0 = uninoculated control; B1 = seed priming (SP); B2 = seed priming + bacterial culture filtrate foliar application (SP + BCF) and F0 = 0% RDF (recommended dose of fertilizer); F1 = 50% RDF; F2 = 75% RDF; F3 = 100% RDF.

Bacterial application increased chlorophyll contents in rice (Figure 2). The highest *chl a*, *chl b*, total chlorophyll, and SPAD values were recorded in F3B2, followed by F3B1 (Figure 2A–D). Chlorophyll contents decreased with reduced fertilizer, but PR5 consistently enhanced the production of chlorophyll at all fertilizer levels compared to the respective uninoculated control. SP and SP + BCF showed similar effects at 50%–100% RDF, while BCF addition further improved chlorophyll at 0% RDF and boosted SPAD values at all levels. Notably, F2B2 (75% RDF + PR5) surpassed 100% RDF (F3B0), with 16.8% more *chl a*, 10.7% more *chl b*, 15% more total chlorophyll, and 1.6% higher SPAD.

### 3.3 Influence of Bacterial Inoculation on Root Growth and Development of Rice

**FIGURE 3:**
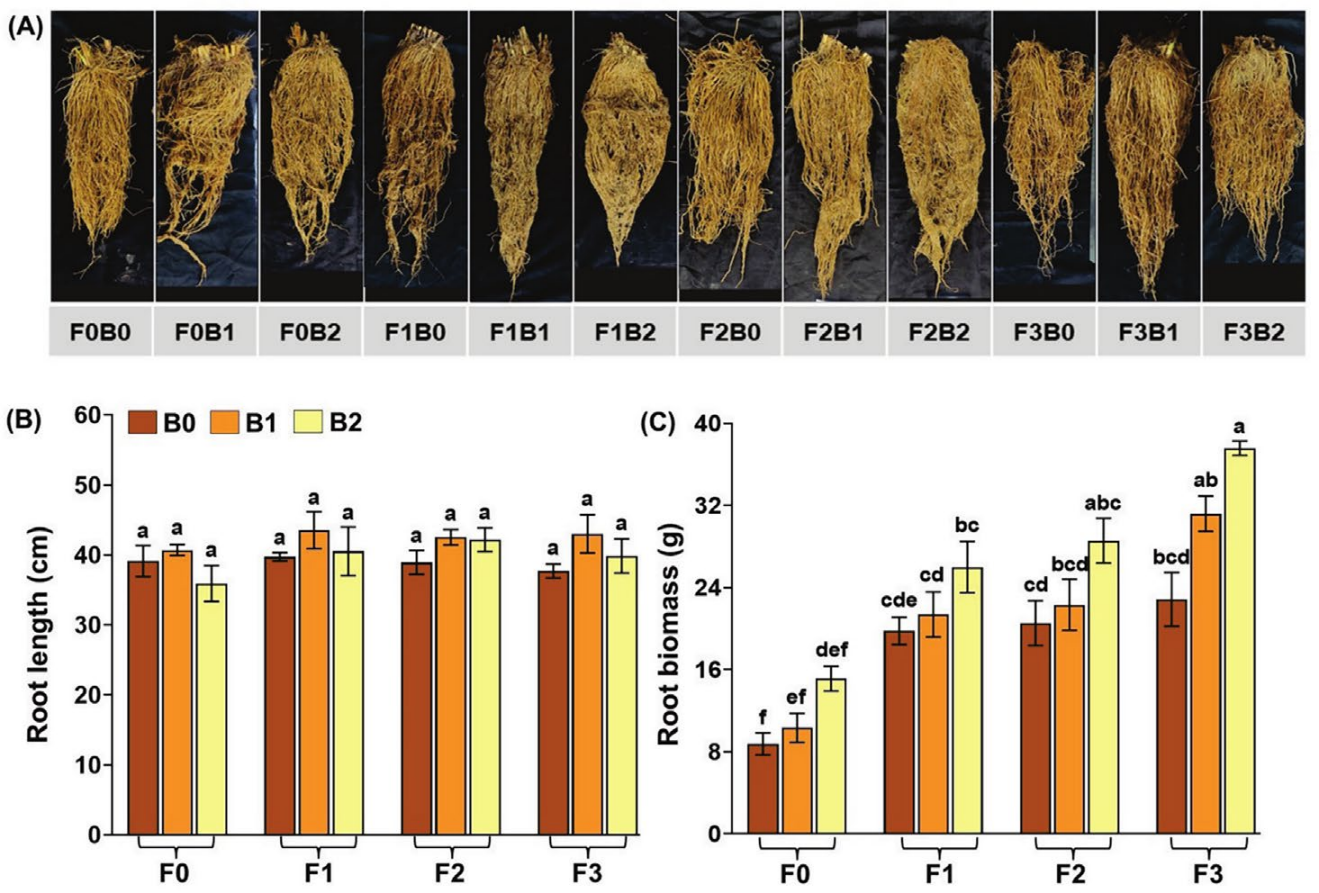
Effect of *Pseudomonas mosselii* PR5 on root structure of BRRI dhan29. (A) photographic view of post‐harvest roots, (B) root length, and (C) root dry biomass. Bars (mean ± standard error, *n* = 3) with similar letters are not significantly differed according to Tukey's test (*p* < 0.05). B0 = uninoculated control; B1 = seed priming (SP); B2 = seed priming + bacterial culture filtrate foliar application (SP + BCF); F0 = 0% RDF (recommended dose of fertilizer); F1 = 50% RDF; F2 = 75% RDF; F3 = 100% RDF.

Similar to the aboveground growth, the root growth and development were significantly influenced by bacterial inoculation (Figure 3). PR5 application increased root biomass across all fertilizer levels, with F3B2 showing a 64.5% increase in root dry biomass over F3B0 (Figure 3C). Importantly, F2B2 treatment produced 39.1% more dry root biomass than its respective controls and even surpassed the 100% RDF (F3B0) by 25.1%. Bacterial treatments promoted lateral and fibrous root proliferation, forming dense, mat‐like structures (Figure 3A). Nevertheless, there was no significant alteration found in root length, and all treatments were found to be statistically similar (Figure 3B).

### 3.4 Effect of PR5 Application on Yield and Yield Attributes of Rice Under Reduced Rate of Fertilizer Application

**FIGURE 4:**
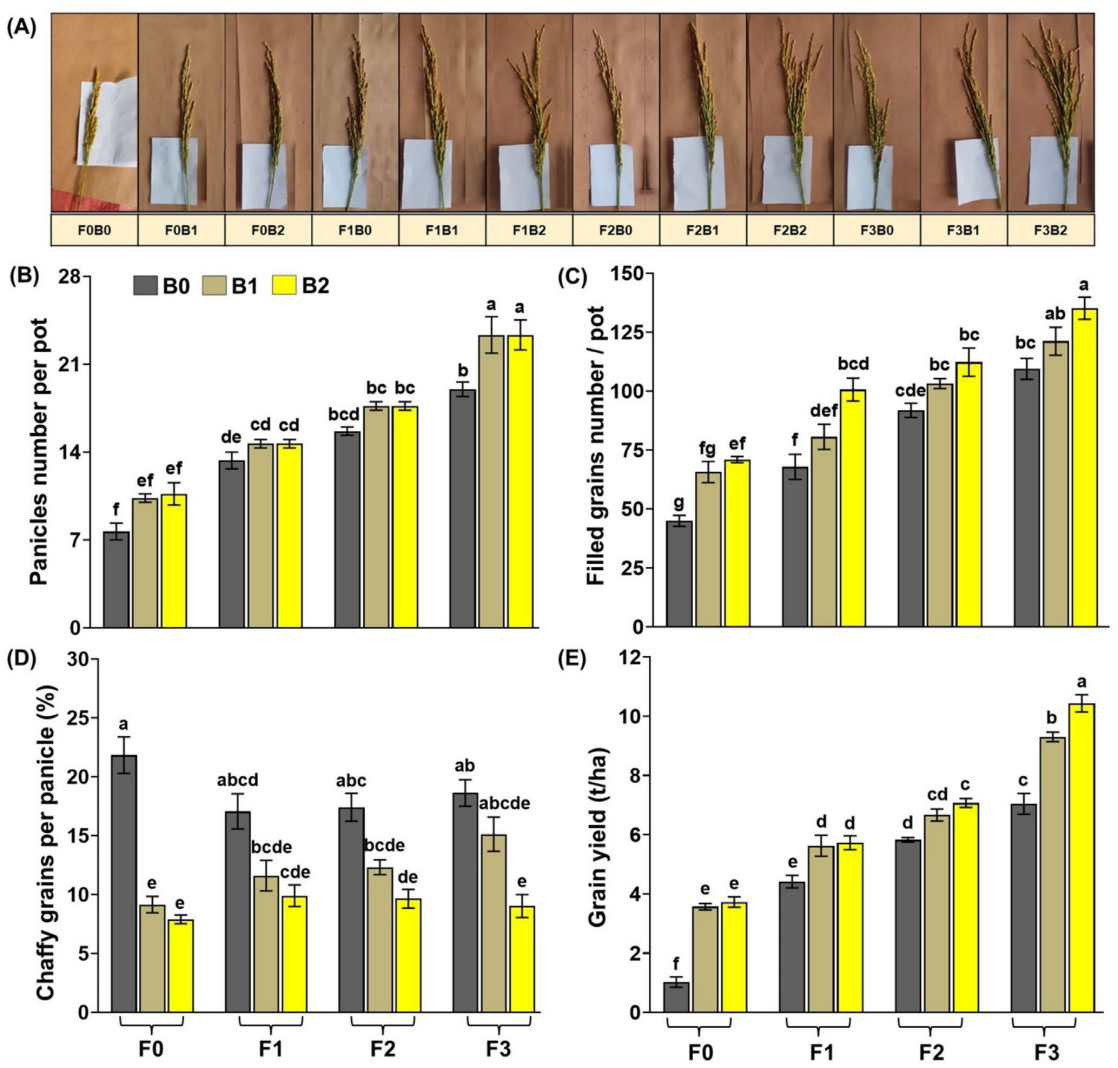
Effect of *Pseudomonas mosselii* PR5 on yield and yield attributes of BRRI dhan29. (A) A comparison photograph of panicle of rice, (B) panicle numbers/pot, (C) filled grains/panicle, (D) chaffy grain (%) and (E) grain yield. Bars (mean ± standard error, *n* = 3) with similar letters are not significantly differed according to Tukey's test (*p* < 0.05). B0 = uninoculated control; B1 = seed priming (SP); B2 = seed priming + bacterial culture filtrate foliar application (SP + BCF) and F0 = 0% RDF (recommended dose of fertilizer); F1 = 50% RDF; F2 = 75% RDF; F3 = 100% RDF.

Figures 4 and S1 showed that PR5 application significantly influenced the grain yield and yield attributes (panicle length, number of panicles per pot, total grain weight per pot, number of filled grains per pot, and % chaffy grains per pot) in all fertilizer doses. Figure 4A showed the pictorial view of the panicle development of rice at post‐harvest condition. With an optimum supply of fertilizer, F3B2 produced the highest grain weight (g/pot), which ultimately resulted in a 48% increase in yield compared to F3B0 (Figures S1B and 4E). This treatment also remained the top performer in most cases of yield attributes and the least in the case of chaffy grain percentage (Figure 4B–D). Although yield attributes were decreased in response to the reduced rates of fertilizers, PR5 incorporation significantly lifted the parameters compared to the respective uninoculated control. At 75% RDF, PR5 significantly reduced the chaffy grain, increased the panicle length (Figure S1A), recovered the grain yield loss compared to 100% RDF, and produced statistically similar yield in F2B2 (7.07 t/ha) with F3B0 (7.05 t/ha).

### 3.5 Effect of PR5 on Nutrient Contents of Rice Under Nutrients Deficit Condition

**FIGURE 5:**
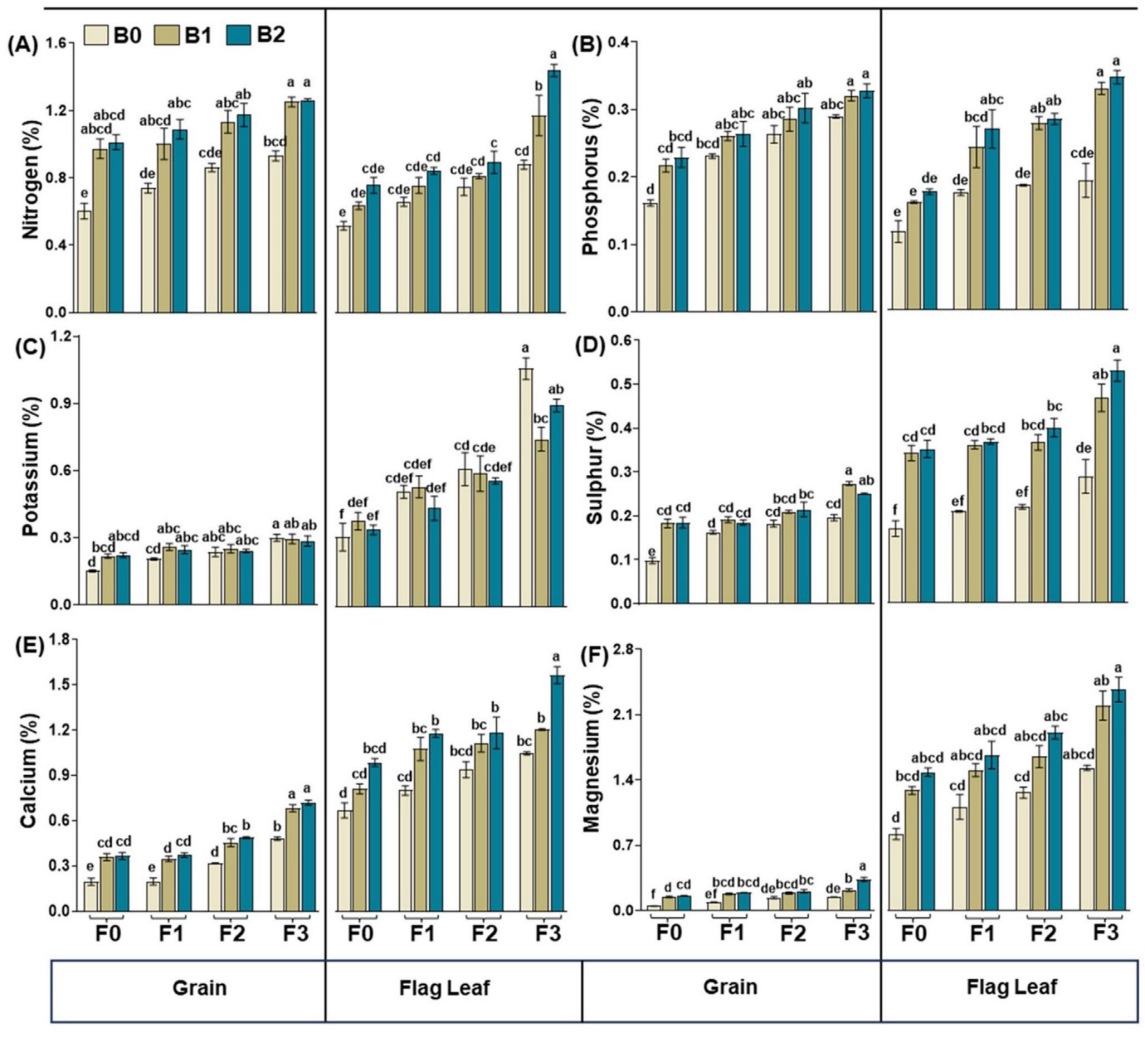
Effect of *Pseudomonas mosselii* PR5 on macronutrients in grain and flag leaf of BRRI dhan29. (A) nitrogen, (B) phosphorus, (C) potassium, (D) sulfur, (E) calcium, and (F) magnesium. Bars (mean ± standard error, *n* = 3) with similar letters are not significantly differed according to Tukey's test (*p* < 0.05). B0 = uninoculated control; B1 = seed priming (SP); B2 = seed priming + bacterial culture filtrate foliar application (SP + BCF) and F0 = 0% RDF (recommended dose of fertilizer); F1 = 50% RDF; F2 = 75% RDF; F3 = 100% RDF.

**FIGURE 6:**
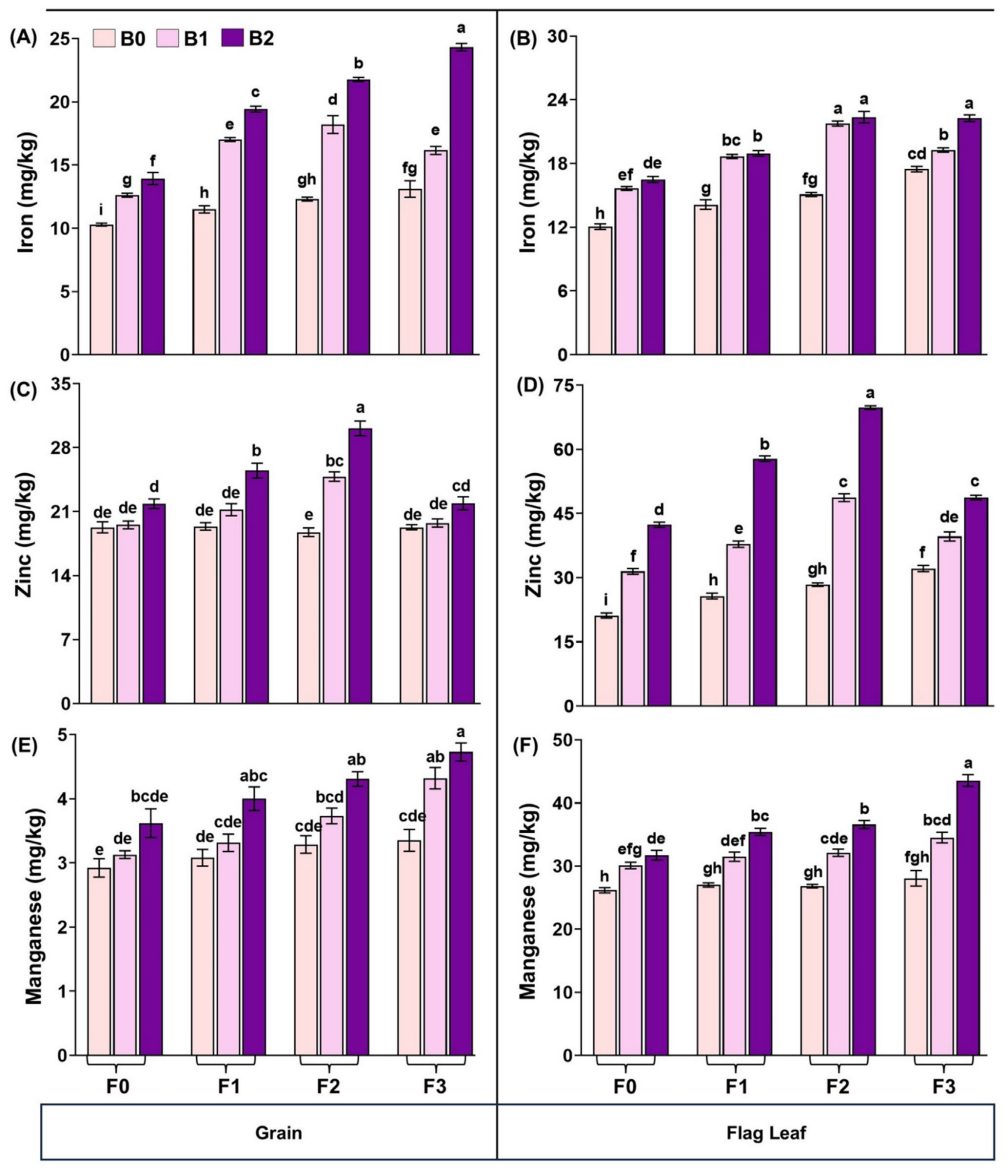
Effect of *Pseudomonas mosselii* PR5 on micronutrients in grain and flag leaf of BRRI dhan29. (A) grain iron, (B) flag leaf iron, (C) grain zinc, (D) flag leaf zinc, (E) grain manganese, and (F) flag leaf manganese. Bars (mean ± standard error, *n* = 3) with similar letter are not different significantly according to Tukey's test (*p* < 0.05). B0 = uninoculated control; B1 = seed priming (SP); B2 = seed priming + bacterial culture filtrate foliar application (SP + BCF) and F0 = 0% RDF (recommended dose of fertilizer); F1 = 50% RDF; F2 = 75% RDF; F3 = 100% RDF.

PR5 treatment improved macronutrient and micronutrient concentrations in both flag leaves and grains under all fertilizer levels (Figures 5 and 6). Although nutrient accumulation declined with reduced fertilizer, PR5 significantly recovered these losses. At 100% RDF, SP and SP + BCF produced the highest and statistically similar levels of N, P, and Ca, whereas F3B2 had the highest Mg and F3B1 the highest S in the grains. Under fertilizer stress, PR5 markedly improved nutrient uptake; for example, F2B2, F1B2, and F0B2 increased grain N by 36.3%–68.1%, P by 14.2%–41.8%, Ca by 53.6%–87.5%, and Mg by 50.8%–221.1% compared to controls. Flag leaf nutrients followed similar trends, with F3B2 showing maximum accumulation. K content increased mainly at 0% and 50% RDF (Figure 5C). For micronutrients, F3B2 maximized Fe and Mn, while F2B2 showed the highest Zn (Figure 6A–F). Compared to controls, PR5 boosted grain Fe (35.3%–76.7%), Zn (13.4%–60.7%), and Mn (23.8%–31%), with even greater gains in flag leaves (up to 145% Zn and 48.2% Fe).

### 3.6 Effect of PR5 on Grain Proximate Composition of Rice Under Nutrients Deficit Condition

**FIGURE 7:**
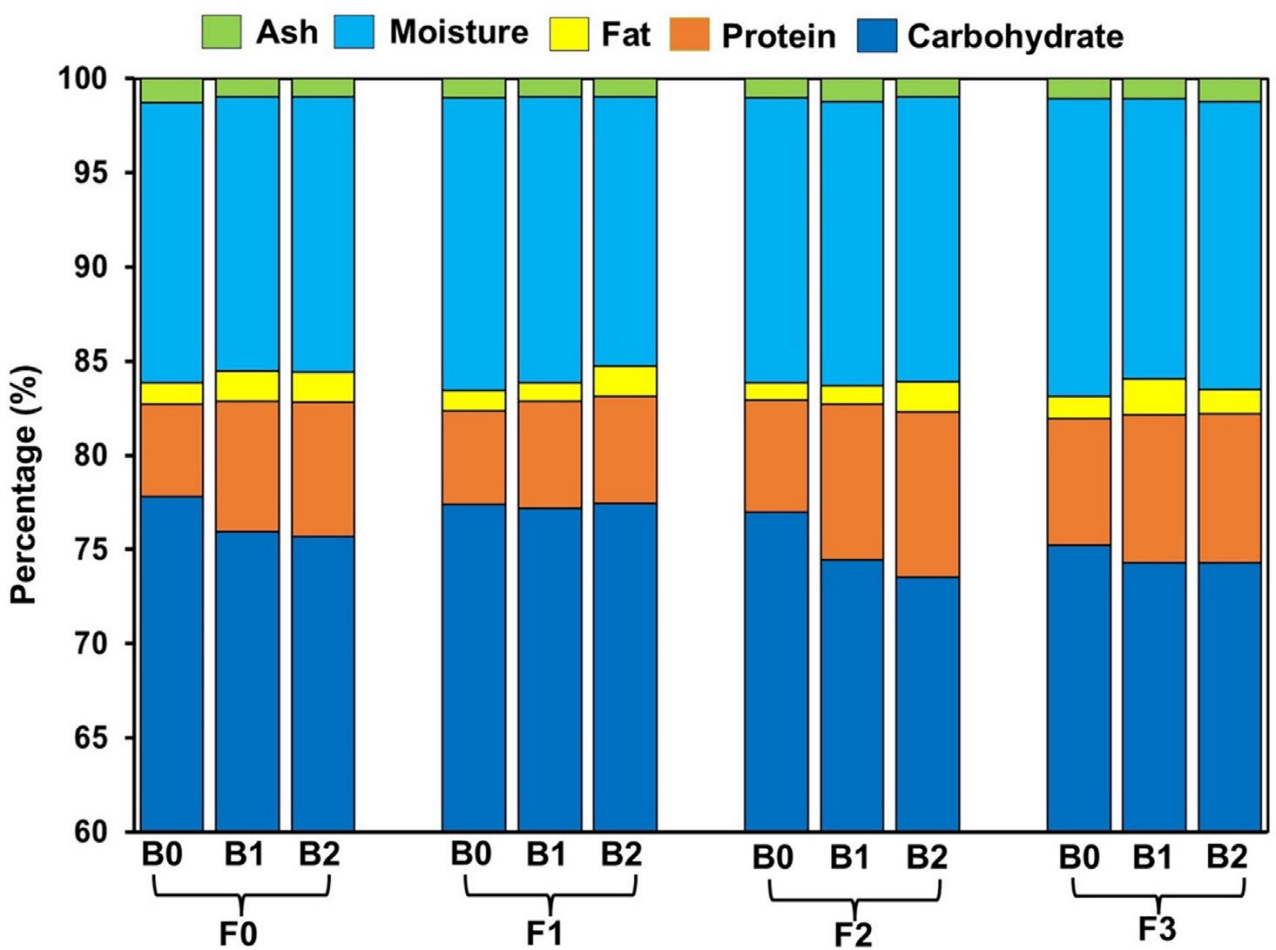
Effect of *Pseudomonas mosselii* PR5 on proximate compositions in grain of BRRI dhan29. B0 = uninoculated control; B1 = seed priming (SP); B2 = seed priming + bacterial culture filtrate foliar application (SP + BCF) and F0 = 0% RDF (recommended dose of fertilizer); F1 = 50% RDF; F2 = 75% RDF; F3 = 100% RDF.

To assess grain quality, grain proximate analysis was done, and it revealed a significant influence by PR5 application (Figure 7). Bacterial‐inoculated treatments produced better proximate composition with higher protein, fat, and ash contents. The maximum protein content (7.9%) and ash content (1.2%) were produced by the F3B2, and F3B1 produced the highest fat content (1.9%) compared to the respective control. Noticeably, despite receiving 25% less fertilizer, F2B2 increased the protein, fat, and ash contents by 13.9%, 7.3%, and 7.6%, respectively, compared to the conventional method (F3B0). Conversely, the carbohydrate content was found to be higher in the control receiving no bacteria in each fertilizer group.

### 3.7 Effect of PR5 on the Availability of Post‐Harvest Soil Nutrients

**FIGURE 8:**
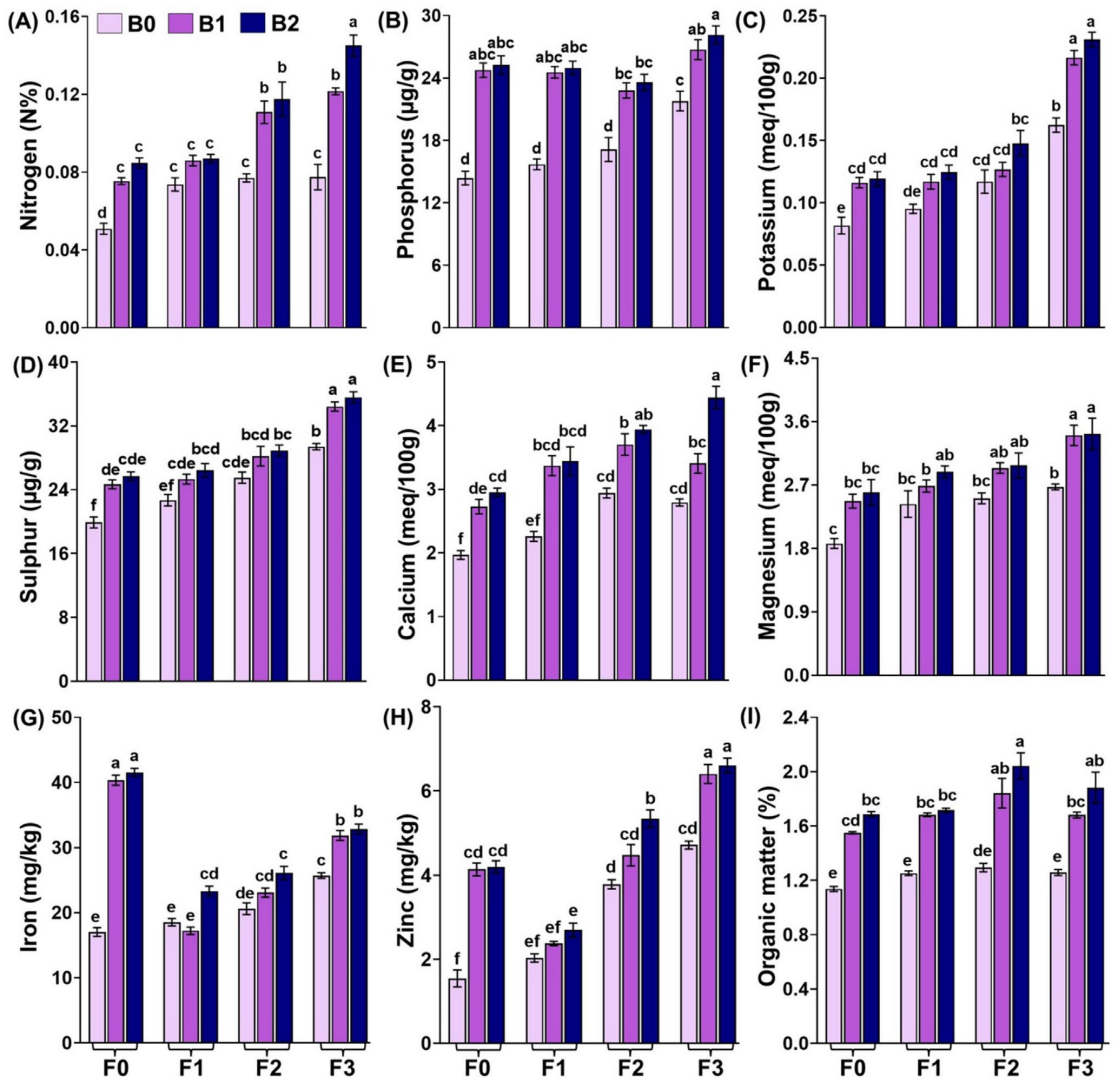
Effect of *Pseudomonas mosselii* PR5 on post‐harvest soil nutrients. (A) nitrogen, (B) phosphorus, (C) potassium, (D) sulfur, (E) calcium, (F) magnesium, (G) iron, (H) zinc, and (I) organic matter. Bars (mean ± standard error, *n* = 3) with similar letters are not significantly different according to Tukey's test (*p* < 0.05). B0 = uninoculated control; B1 = seed priming (SP); B2 = seed priming + bacterial culture filtrate foliar application (SP + BCF); F0 = 0% RDF (recommended dose of fertilizer); F1 = 50% RDF; F2 = 75% RDF; F3 = 100% RDF.

Figure 8 illustrates that PR5 application markedly improved post‐harvest soil nutrient availability. The highest overall enrichment was observed in F3B2 (100% RDF + PR5). Even under reduced fertilizer input, PR5 significantly enhanced soil fertility. At 75% RDF (F2B2), soil N, P, K, Ca, Mg, Fe, and Zn increased by 13%–53% compared to the control (Figure 8A–H). At 50% RDF (F1B2), improvements were notable for P (58.9%), Ca (52.2%), and other nutrients ranging from 16% to 32%. Remarkably, at 0% RDF (F0B2), PR5 greatly boosted soil nutrients, including 66.5% higher N, 75.6% P, 45.8% K, and more than 140% Fe and 170% Zn over the absolute control. In addition, PR5 consistently increased soil organic matter by 37%–50% across all fertilizer levels (Figure 8I).

### 3.8 Effect of *P. mosselii* PR5 on Profitability of Rice at Reduced Rate of Fertilizer Application

Application of PR5 significantly increased the benefit–cost ratio of rice production, making the cultivation more productive and economic (Table 2). When the fertilizer dose was reduced from 100% RDF (F3B0) to 75% (F2B0), the BCR dropped by 29.09%. Nevertheless, adding PR5 not only recovered this loss but also increased the BCR in each group of fertilizers compared to their control. When PR5 was applied with 75% RDF as SP and SP + BCF (F2B1 and F2B2 treatments), the BCR increased by 21.27% and 31.38%, respectively, compared to F2B0 (uninoculated control at 75% RDF condition). It is worth noting that, under 75% RDF, B2 treatment produced a higher result (11.63% increase), and B1 treatment showed a statistically similar result (3.03% increase) than F3B0 treatment (no bacteria at 100% RDF). The largest increase in BCR (207.91%) was found in F0B2 (0% RDF with SP + BCF) compared to F0B0 (no bacteria in 0% RDF condition).

**TABLE 2:** Effect of *Pseudomonas mosselii* PR5 on economic analysis using different fertilizer doses.

Fertilizer doses	Bacterial application	Treatments	Benefit cost ratio (BCR)
100%	No Bacteria	F3 B0	1.73 ± 0.19^bc^
100%	SP	F3 B1	2.59 ± 0.06^a^
100%	SP + BCF	F3 B2	2.90 ± 0.11^a^
75%	No Bacteria	F2 B0	1.23 ± 0.07^cd^
75%	SP	F2 B1	1.78 ± 0.02^bc^
75%	SP + BCF	F2 B2	1.93 ± 0.05^b^
50%	No Bacteria	F1 B0	0.84 ± 0.1d^e^
50%	SP	F1 B1	1.43 ± 0.13^c^
50%	SP + BCF	F1 B2	1.47 ± 0.08^c^
0%	No Bacteria	F0 B0	(−) 0.46 ± 0.07^f^
0%	SP	F0 B1	0.43 ± 0.08^e^
0%	SP + BCF	F0 B2	0.50 ± 0.08^**e**^

*Note:* Values are presented as mean ± standard error mean (*n* = 3), and values with the same superscript letter within the column do not differ significantly according to Tukey's test (*p* < 0.05) (F3 = 100% RDF; F2 = 75% RDF; F1 = 50% RDF; F0 = 0% RDF and B0 = uninoculated control; B1 = seed priming (SP); B2 = seed priming + bacterial culture filtrate foliar application (SP + BCF)). The exchange rate provided in the Materials and Methods section was employed for currency conversion during BCR calculation.

### 3.9 Hierarchical Clustering and Principal Component Analysis of the Data

**FIGURE 9:**
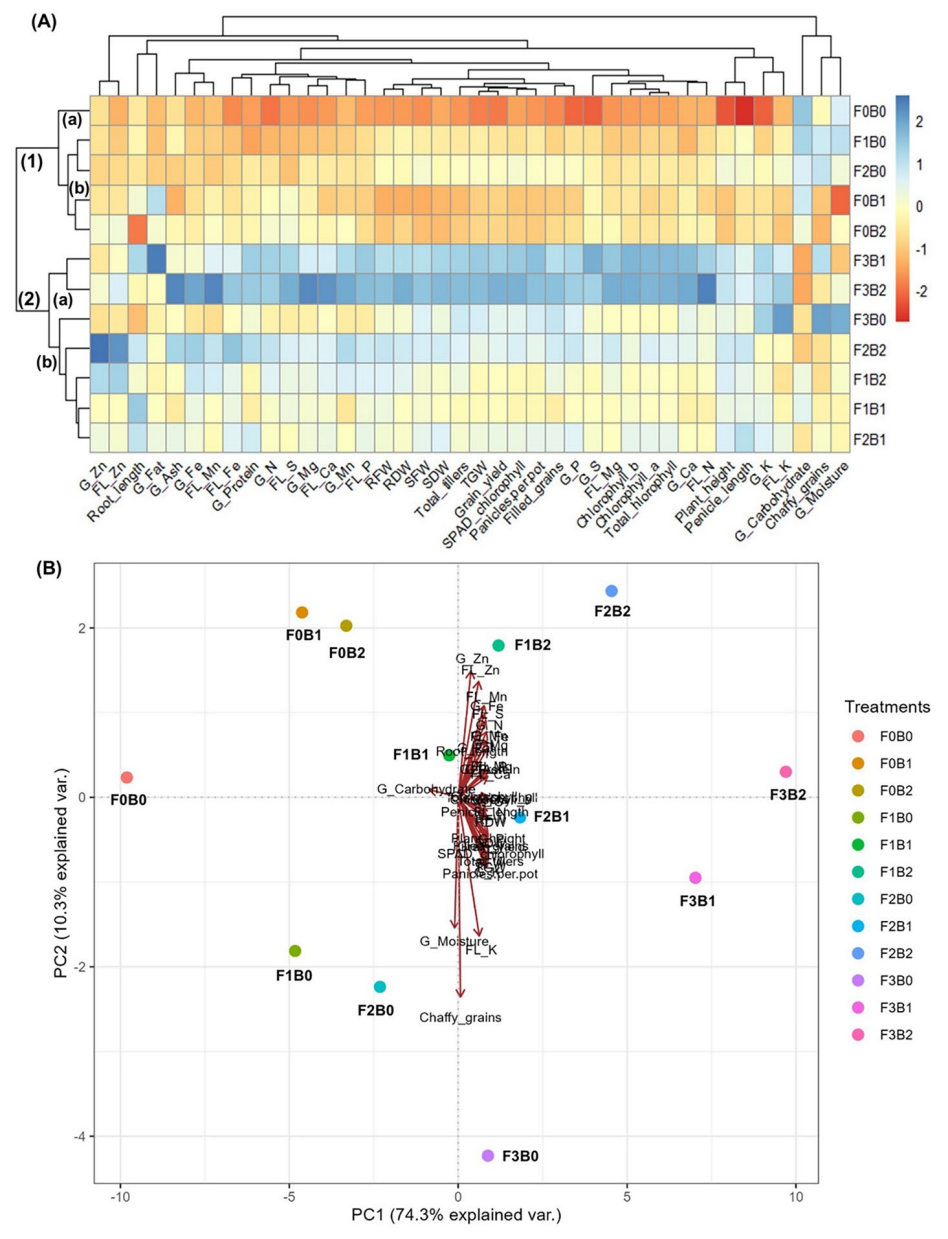
Hierarchical clustering (A) and principal component analysis of plant parameters (B) of BRRI dhan29 at different treatments. B0 = uninoculated control; B1 = seed priming (SP); B2 = seed priming + bacterial culture filtrate foliar application (SP + BCF) and F0 = 0% RDF (recommended dose of fertilizer); F1 = 50% RDF; F2 = 75% RDF; F3 = 100% RDF.

**FIGURE 10:**
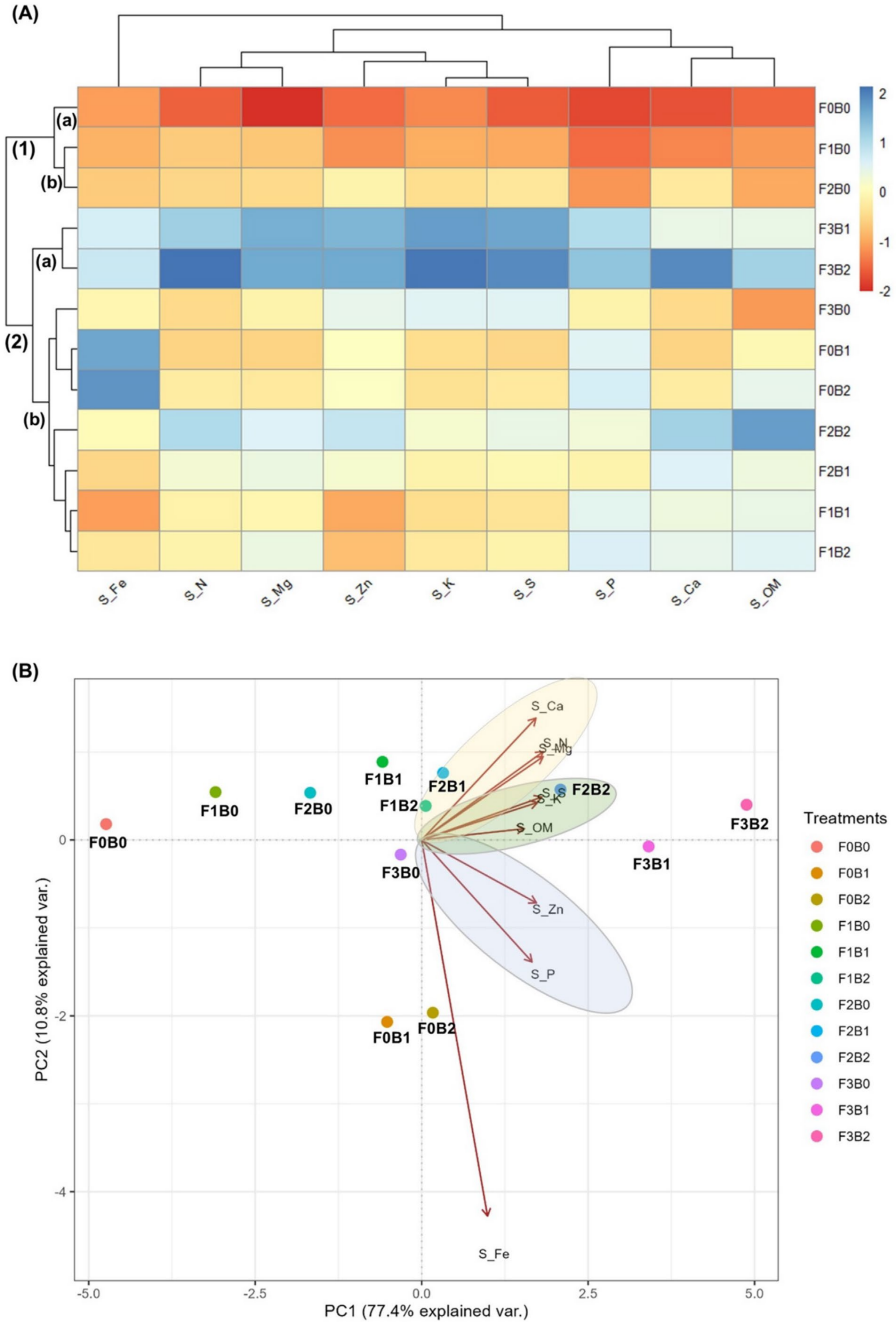
Hierarchical clustering (A) and principal component analysis (B) of soil parameters after growing BRRI dhan29 at different treatments. B0 = uninoculated control; B1 = seed priming (SP); B2 = seed priming + bacterial culture filtrate foliar application (SP + BCF) and F0 = 0% RDF (recommended dose of fertilizer); F1 = 50% RDF; F2 = 75% RDF; F3 = 100% RDF.

Hierarchical clustering grouped treatments into two major clusters for both plant traits (Figure 9A) and soil nutrients (Figure 10A). For plant parameters, cluster I included mainly low‐fertilizer and uninoculated treatments (F0B0, F1B0, F2B0, F0B1, F0B2), while cluster II comprised the remaining treatments, with F3B1–F3B2 showing strong positive correlations and F0B0 negative associations. Grain and flag leaf Zn were strongly linked with F2B2. A similar clustering trend was observed for soil nutrients. Cluster 1 (F0B0, F1B0, F2B0) divided again into F0B0 and F1B0–F2B0, whereas cluster II separated into F3B1–F3B2 and the remaining treatments (F3B0, F0B1, F0B2, F2B2, F2B1, F1B1, F1B2).

PCA confirmed these patterns, with the first two PCs explaining 84.6% of the variation in plant traits (PC1 = 74.3%, PC2 = 10.3%) (Figure 9B). F3B2, F3B1, and F2B2 were strongly associated with most plant traits except grain moisture and carbohydrate, while F1B0 and F2B0 showed negative associations. For soil nutrients, PC1 and PC2 explained 88.2% of variation (Figure 10B), with F3B2, F3B1, and F2B2 positively linked to all nutrients, while low‐fertilizer controls (F2B0, F1B0, F0B0) showed weak or negative associations.

## 4 Discussion

*Pseudomonas* spp. has come under the attention of the researchers in recent times for their extraordinary capability of plant growth promotion in any adverse conditions (Sandhya et al. 2010; Shen et al. 2013). The present study demonstrated the potential of *P. mosselii* PR5 to promote rice growth, improve nutrient use efficiency, and sustain productivity under sub‐optimal chemical fertilizer conditions. The multifaceted benefits observed highlight the potentiality of PR5 as a bioinoculant to reduce chemical fertilizer dependency without compromising yield or grain quality, which is discussed below.

### 4.1 Promotion of Rice Growth and Photosynthetic Efficiency Under Reduced Fertilizer Conditions

Application of PR5 with different fertilizer doses significantly enhanced rice growth traits, including plant height, tiller number, chlorophyll content, and biomass, demonstrating strong efficacy under nutrient‐deficient conditions. These effects are mediated through multiple mechanisms: nutrient uptake via N fixation, phytohormone (IAA) synthesis, and solubilization of P, Zn, and Si (de Andrade et al. 2023; Paungfoo‐Lonhienne et al. 2019; Ríos‐Ruiz et al. 2025; Thammasittirong et al. 2025). PR5 also produces siderophores and HCN, which support iron acquisition, disease suppression, and pathogen inhibition and induce systemic resistance in plants under biotic stress and thereby increase plant growth (Haas and Défago 2005; Ahmed and Holmström 2014). Increased photosynthetic pigments suggest better chloroplast function under nutrient stress (Sun et al. 2024). Moreover, seed priming stimulated lateral and fibrous root development, while BCF foliar application further promoted root proliferation through IAA production (Glick 2014), improving soil exploration, nutrient uptake, and water absorption (Liu et al. 2017). The improvements in both above‐ and below‐ground growth parameters confirmed that PR5 is particularly effective under limited fertilizer conditions, offering a promising strategy for resource‐efficient rice production.

### 4.2 Enhancement of Yield Attributes and Yield of Rice Under Reduced Fertilizer Conditions

The role of PGPR, particularly *Pseudomonas*, in yield improvement has been widely reported (Chandra and Sharma 2021; Elekhtyar et al. 2022; Kumawat et al. 2019; Xiao et al. 2020; Yasmin et al. 2016). In this study, PR5 seed priming significantly improved yield attributes‐including panicle number, filled grains, and overall yield across all fertilizer levels, with further enhancement under BCF foliar application. Notably, F2B2 (75% RDF + SP + BCF) achieved yields comparable to or exceeding the conventional 100% RDF (F3B0), indicating that fertilizer input can be reduced by 25% without yield loss. This effect may be linked to higher production of growth‐promoting biomolecules under stress (Balasjin et al. 2023; Cheng et al. 2012; Rajkumar et al. 2017; Tanveer et al. 2023). PR5 likely improved reproductive success through better nutrient partitioning and hormone‐mediated panicle development (Grover et al. 2011; Adesemoye et al. 2009). Additionally, enhanced photosynthetic activity and improved root‐mediated nutrient and water uptake collectively lead to higher yields.

### 4.3 Improvement of Nutrient Contents and Grain Quality Under Reduced Fertilizer Conditions

The study revealed that PR5 markedly enhanced macro‐ and micronutrient uptake in rice flag leaves and grains, especially under nutrient‐deficient conditions, and positively influenced grain proximate composition. PR5 promotes root branching and root hair development, increasing root surface area for nutrient absorption and facilitating efficient transfer from roots to shoots (Glick 2012). It enhances nitrogen uptake through stimulation of root growth and microbial N cycling (Bhattacharyya and Jha 2012), and secretes organic acids, enzymes, and siderophores that solubilize phosphorus, zinc, potassium, and iron, improving their bioavailability (Ahmed and Holmström 2014; Rodríguez and Fraga 1999; Zaidi et al. 2009). Absorbed nutrients are transported via xylem to metabolically active tissues such as the flag leaf, contributing to grain filling (Marschner 2012). Increased nitrogen uptake by PR5 inoculated rice plant enhance the assimilation of nitrogen in rice grain which improved grain protein content as suggested by Turino Mattos et al. (2023). Furthermore, rhizobacterial phytohormones enhance xylem vessel development and phloem loading efficiency, promoting nutrient mobilization, grain protein synthesis, and overall grain nutritional quality (Spaepen 2014).

### 4.4 Restoration of Soil Health and Nutrient Recycling

Post‐harvest soil nutrient availability and organic matter were greatly enhanced by PR5 inoculation. PR5 improves soil nitrogen mainly through ammonification and associative nitrogen fixation (Ahmad et al. 2008) and solubilizes phosphorus by secreting organic acids such as gluconic, citric, and oxalic acids, which chelate cations like Ca^2+^, Fe^3+^, and Al^3+^, releasing phosphate and zinc (Rodríguez and Fraga 1999; Ramesh et al. 2014; Sharma et al. 2013). It also increases soluble K^+^ via organic acid generation and acidolysis (Basak and Biswas 2010; Meena et al. 2016) and improves the sulfur cycle by oxidizing elemental sulfur (Ranadev et al. 2023). Acidification mobilizes Ca^2+^ and Mg^2+^ from carbonates and silicates (Dakora and Phillips 2002), while siderophore production makes Fe^3+^ bioavailable (Ahmed and Holmström 2014). Complex organic matter can be converted to simple nutrients by hydrolytic enzymes secreted by PR5, which include β‐glucosidase, cellulase, proteases, lipase, and chitinase (Khatoon et al. 2017; Vyas and Gulati 2009), and the labile carbon can be released into the rhizosphere by PR5 (Singh et al. 2022). The enzymes are responsible for carbon cycling and break cellulose and other carbohydrates down to enable the decomposition of soil organic matter (Wang and Xu 2026). Microbial decomposition of plant residue transforms carbon input into soil organic carbon (SOC), which together with biomass turnover and necromass formation are the key contributors to soil organic carbon formation (Chandel et al. 2023; Philippot et al. 2024; Wu et al. 2024). The mechanisms ultimately improve soil structure, fertility, water holding capacities, and cation exchange capacity, which leads to long‐term soil health.

### 4.5 Economic Feasibility and Profitability

The current study revealed that PR5 not only promoted plant growth and development but also enhanced the economic profitability of rice production. The bacterium directly improved yield and benefit–cost ratio by stimulating growth, enhancing nutrient acquisition, improving grain quality, and restoring soil health. PR5 reduced the need for chemical fertilizers by up to 25% while maintaining yield, lowering production costs, and increasing profitability. Supporting evidence includes Pame et al. (2023), who reported that PGPR increased net income by 79% and BCR by 23% while reducing production costs by 19%. Likewise, Turino Mattos et al. (2023) reported that *A. brasilense* + *P. fluorescens* increased rice output by 37%. Furthermore, PR5 contributes to long‐term soil health, potentially reducing dependence on chemical pesticides and fertilizers and offering sustained economic benefits.

### 4.6 Implications for Food Security, Nutritional Outcomes, and Environmental Health

Considering the increasing demand for food worldwide, a sustainable approach to crop production is essential, particularly for nations with dense populations. Bio‐inoculation of rice with *P. mosselii* PR5 offers a promising approach, enhancing plant growth, yield, grain quality, and soil fertility. PR5 also improved grain nutritional value, increasing protein, fat, and micronutrients (Fe, Zn), supporting health and long‐term food security. Importantly, PR5 performed well under nutrient‐deficient conditions, allowing a 25% reduction in chemical fertilizers without yield loss, making rice production economically viable. The strain also acts as a biocontrol agent against rice blast (Sultana et al. 2024b, 2024c), reducing reliance on pesticides and thereby decreasing the overall agrochemical use. Consistent with previous studies (Liu et al. 2017; Odoh et al. 2020), our findings show that integrating PR5 maintained optimal yield and benefit–cost ratios while minimizing chemical inputs. Lower agrochemical use ultimately reduces risk on human health, while reducing environmental impacts, including soil degradation, water pollution, and biodiversity loss.

From a practical standpoint, the choice depends on production goals: if the priority is maximum yield, combining seed priming with PR5 bacterial culture filtrate foliar application and a full fertilizer dose is recommended. However, if we want to sustain our ecosystem, it would be better to reduce at least 25% of the recommended dose of fertilizer while retaining the optimal yield. In summary, integrating PR5 into rice cultivation provides agronomic, nutritional, and environmental benefits, promoting climate‐smart, sustainable agriculture that balances food security with ecosystem protection.

## 5 Conclusion

This study conclusively demonstrates that *P. mosselii* strain PR5 significantly promotes plant growth and development, offering both economic and environmental advantages in rice production. PR5 not only enhances plant performance under optimal conditions but also supports resilience and sustained productivity in nutrient‐stressed environments. Among the tested treatments, the combination of PR5 (SP + BCF) with 100% of the recommended dose of fertilizer (RDF) resulted in approximately a 50% increase in grain yield. However, despite this yield boost, the continued reliance on a full dose of chemical fertilizer raises concerns about agricultural pollution and its long‐term environmental impacts. More notably, the combination of PR5 (SP + BCF) with a reduced fertilizer application (75% RDF) demonstrated the potential to lower chemical fertilizer use by 25% without compromising crop performance. This treatment produced comparable grain yields, improved nutrient uptake, and enhanced grain quality and proximate composition while maintaining soil fertility. It thereby supports a more sustainable approach to rice cultivation. Economic analysis further validated the benefits of this approach, indicating a higher benefit–cost ratio compared to conventional practices. Overall, the integration of PR5 biopriming with reduced fertilizer input presents a promising strategy for enhancing agricultural productivity, profitability, ecological sustainability, and restoration of environmental health.

## Funding

This work was supported by the Ministry of Education, Government of the People's Republic of Bangladesh (Grant LS20211677).

## Ethics Statement

The authors have nothing to report.

## Consent

The authors have nothing to report.

## Conflicts of Interest

The authors declare no conflicts of interest.

## Supporting information


**Table S1:** Physio‐chemical properties of initial soil.
**Table S2:** Monthly average weather data for the experimental period (January–June 2024) obtained from the Bangladesh Agricultural University (BAU) Meteorological Station.
**Table S3:** Summary of two‐way ANOVA results showing the effects of fertilizer level (F), bacterial inoculation (B), and their interaction (F × B) on plant growth, yield, grain nutritional quality, nutrient uptake, and soil physicochemical properties. F‐statistics and corresponding *p* values are presented for each variable.
**Figure S1:** Effect of *P. mosselii* PR5 on (A) panicle length and (B) total grain weight of BRRI dhan29. Bars (mean ± standard error) with similar letter are not differed significantly according to Tukey's test (*p* < 0.05). B0 = no bacteria; B1 = seed priming (SP); B2 = seed priming + bacterial cell filtrate foliar application (SP + BCF) and F0 = 0% RDF (recommended dose of fertilizer); F1 = 50% RDF; F2 = 75% RDF; F3 = 100% RDF.

## Data Availability

All data supporting the findings of the study are included in the article in tables, figures, and Supporting Information.

## References

[pei370182-bib-0001] Adesemoye, A. O. , H. A. Torbert , and J. W. Kloepper . 2009. “Plant Growth‐Promoting Rhizobacteria Allow Reduced Application Rates of Chemical Fertilizers.” Microbial Ecology 58, no. 4: 921–929. 10.1007/s00248-009-9531-y. 19466478

[pei370182-bib-0002] Ahmad, F. , I. Ahmad , and M. S. Khan . 2008. “Screening of Free‐Living Rhizospheric Bacteria for Their Multiple Plant Growth Promoting Activities.” Microbiological Research 163, no. 2: 173–181. 10.1016/j.micres.2006.04.001. 16735107

[pei370182-bib-0003] Ahmed, E. , and S. J. Holmström . 2014. “Siderophores in Environmental Research: Roles and Applications.” Microbial Biotechnology 7, no. 3: 196–208. 10.1111/1751-7915.12117. 24576157 PMC3992016

[pei370182-bib-0004] Ahmmed, S. , M. Jahiruddin , S. Razia , et al. 2018. Fertilizer Recommendation Guide‐2018, 223. Bangladesh Agricultural Research Council (BARC). https://www.bfa‐fertilizer.org/wp‐content/uploads/2019/09/Fertilizer‐Recommendation‐Guide‐2018‐English.pdf.

[pei370182-bib-0005] Ajmal, M. , H. I. Ali , R. Saeed , et al. 2018. “Biofertilizer as an Alternative for Chemical Fertilizers.” Journal of Agriculture and Allied Sciences 7, no. 1: 1–7.

[pei370182-bib-0006] AOAC . 2004. Official Methods of Analysis. 15th ed. Association of Official Analytical Chemists. 10.12691/ajfst-2-4-4.

[pei370182-bib-0007] Arnon, D. I. 1949. “Copper Enzymes in Isolated Chloroplasts. Polyphenol Oxidase in *Beta vulgaris* .” Plant Physiology 24: 1–15. 10.1104/pp.24.1.1. 16654194 PMC437905

[pei370182-bib-0008] Balasjin, N. M. , J. S. Maki , and M. R. Schläppi . 2023. “ *Pseudomonas mosselii* Improves Cold Tolerance in Rice.” Canadian Journal of Microbiology 70, no. 1: 15–31. 10.1139/cjm-2023-0030. 37699259

[pei370182-bib-0009] Basak, B. B. , and D. R. Biswas . 2010. “Co‐Inoculation of Potassium Solubilizing and Nitrogen Fixing Bacteria on Solubilization of Waste Mica and Their Effect on Growth Promotion and Nutrient Acquisition by a Forage Crop.” Biology and Fertility of Soils 46, no. 6: 641–648. 10.1007/s00374-010-0456-x.

[pei370182-bib-0010] Bhattacharyya, P. N. , and D. K. Jha . 2012. “Plant Growth‐Promoting Rhizobacteria (PGPR): Emergence in Agriculture.” World Journal of Microbiology and Biotechnology 28, no. 4: 1327–1350. 10.1007/s11274-011-0979-9. 22805914

[pei370182-bib-0011] Black, C. A. , D. D. Evans , L. E. Ensminger , and J. L. White . 1965. Methods of Soil Analysis. American Society of Agronomy. 10.2134/agronmonogr9.1.

[pei370182-bib-0012] Bremner, J. M. 1965. “Total Nitrogen.” In Methods of Soil Analysis. Part 2, edited by C. A. Black , 2nd (ed.) ed., 1149–1178. ASA. 10.2134/agronmonogr9.2.c32.

[pei370182-bib-0013] Chandel, A. K. , L. Jiang , and Y. Luo . 2023. “Microbial Models for Simulating Soil Carbon Dynamics: A Review.” Journal of Geophysical Research: Biogeosciences 128, no. 8: e2023JG007436. 10.1029/2023JG007436.

[pei370182-bib-0014] Chandra, D. , and A. K. Sharma . 2021. “Field Evaluation of Consortium of Bacterial Inoculants Producing ACC Deaminase on Growth, Nutrients and Yield Components of Rice and Wheat.” Journal of Crop Science and Biotechnology 24, no. 3: 293–305. 10.1007/s12892-020-00077-y.

[pei370182-bib-0015] Cheng, Z. , O. Z. Woody , B. J. McConkey , and B. R. Glick . 2012. “Combined Effects of *Pseudomonas putida* UW4 and Salinity Stress on *Brassica napus* .” Applied Soil Ecology 61: 255–263. 10.1016/j.apsoil.2011.10.006.

[pei370182-bib-0016] Chesnin, L. , and C. H. Yien . 1951. “Turbidimetric Determination of Available Sulfates.” Soil Science Society of America Journal 15: 149–151. 10.2136/sssaj1951.036159950015000C0032x.

[pei370182-bib-0017] Chieb, M. , and E. W. Gachomo . 2023. “The Role of Plant Growth Promoting Rhizobacteria in Plant Drought Stress Responses.” BMC Plant Biology 23: 407. 10.1186/s12870-023-04403-8. 37626328 PMC10464363

[pei370182-bib-0018] Costa‐Gutierrez, S. B. , C. Adler , M. Espinosa‐Urgel , and R. E. de Cristóbal . 2022. “ *Pseudomonas putida* and Its Close Relatives: Mixing and Mastering the Perfect Tune for Plants.” Applied Microbiology and Biotechnology 106, no. 9: 3351–3367. 10.1007/s00253-022-11881-7. 35488932 PMC9151500

[pei370182-bib-0019] Dakora, F. D. , and D. A. Phillips . 2002. “Root Exudates as Mediators of Mineral Acquisition in Low‐Nutrient Environments.” Plant and Soil 245, no. 1: 35–47. 10.1023/A:1020809400075.

[pei370182-bib-0020] de Andrade, L. A. , C. H. B. Santos , E. T. Frezarin , L. R. Sales , and E. C. Rigobelo . 2023. “Plant Growth‐Promoting Rhizobacteria for Sustainable Agricultural Production.” Microorganisms 11, no. 4: 1088. 10.3390/microorganisms11041088. 37110511 PMC10146397

[pei370182-bib-0021] Dillon, J. L. , and J. B. Hardaker . 1980. Farm Management Research for Small Farmer Development. Vol. 41. FAO. https://books.google.com.bd/books/about/Farm_Management_Research_for_Small_Farme.html?id=1MDdtAEACAAJ&redir_esc=y.

[pei370182-bib-0022] Egamberdieva, D. , and Z. Kucharova . 2009. “Selection for Root Colonising Bacteria Stimulating Wheat Growth in Saline Soils.” Biology and Fertility of Soils 45: 563–571. 10.1007/s00374-009-0366-y.

[pei370182-bib-0023] Elekhtyar, N. M. , M. Awad‐Allah , and A. A. Zidan . 2022. “Effect of Plant Growth‐Promoting Rhizobacteria and Cyanobacteria on Physico‐Chemical and Cooking Characteristics of Giza 179 Rice Grains.” Egyptian Journal of Agricultural Research 100, no. 1: 22–29. 10.21608/ejar.2022.90424.1134.

[pei370182-bib-0024] FAO . 2004. Rice Is Life. Food and Agriculture Organization of the United Nations.

[pei370182-bib-0025] Farnia, A. , and K. Hasanpoor . 2015. “Comparison Between Effect of Chemical and Biological Fertilizers on Yield and Yield Components in Wheat ( *Triticum aestivum* L.).” Indian Journal of Natural Sciences 5, no. 30: 7792–7800.

[pei370182-bib-0026] Ghosh, A. B. , J. C. Bajaj , R. Hasan , and D. Singh . 1983. Soil and Water Testing Method, 1–48. IARI.

[pei370182-bib-0027] Glick, B. R. 2012. “Plant Growth‐Promoting Bacteria: Mechanisms and Applications.” Scientifica 2012, no. 1: 963401. 10.6064/2012/963401. 24278762 PMC3820493

[pei370182-bib-0028] Glick, B. R. 2014. “Bacteria With ACC Deaminase Can Promote Plant Growth and Help to Feed the World.” Microbiological Research 169, no. 1: 30–39. 10.1016/j.micres.2013.09.009. 24095256

[pei370182-bib-0029] Grover, M. , S. Z. Ali , V. Sandhya , A. Rasul , and B. Venkateswarlu . 2011. “Role of Microorganisms in Adaptation of Agriculture Crops to Abiotic Stresses.” World Journal of Microbiology and Biotechnology 27: 1231–1240. 10.1007/s11274-010-0572-7.

[pei370182-bib-0030] Haas, D. , and G. Défago . 2005. “Biological Control of Soil‐Borne Pathogens by Fluorescent Pseudomonads.” Nature Reviews Microbiology 3, no. 4: 307–319. 10.1038/nrmicro1129. 15759041

[pei370182-bib-0031] Hossain, M. E. , S. Shahrukh , and S. A. Hossain . 2022. “Chemical Fertilizers and Pesticides: Impacts on Soil Degradation, Groundwater, and Human Health in Bangladesh.” In Environmental Degradation, 63–92. Springer. 10.1007/978-3-030-95542-7_4.

[pei370182-bib-0032] Jackson, M. L. 1973. Soil Chemical Analysis. Prentice Hall of India.

[pei370182-bib-0033] Kaushal, M. , and S. P. Wani . 2016. “Plant‐Growth‐Promoting Rhizobacteria: Drought Stress Alleviators to Ameliorate Crop Production in Drylands.” Annals of Microbiology 66: 35–42. 10.1007/s13213-015-1112-3.

[pei370182-bib-0034] Khatoon, H. , P. Solanki , M. Narayan , L. Tewari , J. P. N. Rai , and C. Hina Khatoon . 2017. “Role of Microbes in Organic Carbon Decomposition and Maintenance of Soil Ecosystem.” International Journal of Chemical Studies 5, no. 6: 1648–1656.

[pei370182-bib-0035] Kumawat, K. C. , P. Sharma , A. Sirari , et al. 2019. “Synergism of *Pseudomonas aeruginosa* (LSE‐2) Nodule Endophyte With *Bradyrhizobium* sp. (LSBR‐3) for Improving Plant Growth, Nutrient Acquisition and Soil Health in Soybean.” Journal of Molecular Biology 35: 1–17. 10.1007/s11274-019-2622-0. 30834977

[pei370182-bib-0036] Lassaletta, L. , G. Bille , B. Grizzetti , J. Anglade , and J. Garnier . 2014. “50‐Year Trends in Nitrogen Use Efficiency of World Cropping Systems.” Environmental Research Letters 9: 1–9. 10.1088/1748-9326/9/10/105011.

[pei370182-bib-0037] Lichtenthaler, H. K. , and A. R. Wellburn . 1983. “Determinations of Total Carotenoids and Chlorophylls a and b of Leaf Extracts in Different Solvents.” Biochemical Society Transactions 11: 591–592. 10.1042/bst0110591.

[pei370182-bib-0038] Lindsay, W. L. , and W. A. Norvell . 1978. “Development of a DTPA Soil Test for Zinc, Iron, Manganese and Copper.” Soil Science Society of America Journal 42, no. 3: 421–428. 10.2136/sssaj1978.03615995004200030009x.

[pei370182-bib-0039] Liu, Z. , Q. Rong , W. Zhou , and G. Liang . 2017. “Effects of Inorganic and Organic Amendment on Soil Chemical Properties, Enzyme Activities, Microbial Community and Soil Quality in Yellow Clayey Soil.” PLoS One 12, no. 3: e0172767. 10.1371/journal.pone.0172767. 28263999 PMC5338777

[pei370182-bib-0040] Long, D. H. , F. N. Lee , and D. O. TeBeest . 2000. “Effect of Nitrogen Fertilization on Disease Progress of Rice Blast.” Plant Disease 84, no. 4: 403–409. 10.1094/PDIS.2000.84.4.403. 30841161

[pei370182-bib-0041] Lugtenberg, B. J. , N. Malfanova , F. Kamilova , and G. Berg . 2013. “Microbial Control of Plant Root Diseases.” Molecular Microbial Ecology of the Rhizosphere 1: 575–586. 10.1002/9781118297674.ch54.

[pei370182-bib-0042] Malusá, E. , L. Sas‐Paszt , and J. Ciesielska . 2012. “Technologies for Beneficial Microorganisms Inocula Used as Biofertilizers.” Scientific World Journal 2012: 491206. 10.1100/2012/491206. 22547984 PMC3324119

[pei370182-bib-0043] Marschner, H. 2012. Marschner's Mineral Nutrition of Higher Plants. Academic Press. 10.1016/C2009-0-63043-9.

[pei370182-bib-0044] Meena, V. S. , B. R. Maurya , J. P. Verma , and R. S. Meena , eds. 2016. Potassium Solubilizing Microorganisms for Sustainable Agriculture. Vol. 331. Springer. 10.1007/978-81-322-2776-2.

[pei370182-bib-0045] Milošević, N. A. , J. B. Marinković , and B. B. Tintor . 2012. “Mitigating Abiotic Stress in Crop Plants by Microorganisms.” Zbornik Matice Srpske Za Prirodne Nauke 123: 17–26. 10.2298/ZMSPN1223017M.

[pei370182-bib-0046] Odoh, C. K. , K. Sam , N. Zabbey , et al. 2020. “Microbial Consortium as Biofertilizers for Crops Growing Under the Extreme Habitats.” In Plant Microbiomes for Sustainable Agriculture, 381–424. Springer International Publishing. 10.1007/978-3-030-38453-1_13.

[pei370182-bib-0047] Olsen, S. R. 1954. Estimation of Available Phosphorus in Soils by Extraction With Sodium Bicarbonate. USDA. Report No. 939.

[pei370182-bib-0048] Page, A. L. , ed. 1982. Methods of Soil Analysis. Part 2. Chemical and Microbiological Properties. ASA.

[pei370182-bib-0049] Pame, A. R. P. , D. Vithoonjit , N. Meesang , et al. 2023. “Improving the Sustainability of Rice Cultivation in Central Thailand With Biofertilizers and Laser Land Leveling.” Agronomy 13, no. 2: 587. 10.3390/agronomy13020587.

[pei370182-bib-0050] Paungfoo‐Lonhienne, C. , M. Redding , C. Pratt , and W. Wang . 2019. “PGPR Increase Fertilizer Efficiency and Reduce Nitrogen Loss.” Journal of Environmental Management 233: 337–341. 10.1016/j.jenvman.2018.12.052. 30590263

[pei370182-bib-0051] Philippot, L. , C. Chenu , A. Kappler , M. C. Rillig , and N. Fierer . 2024. “The Interplay Between Microbial Communities and Soil Properties.” Nature Reviews Microbiology 22, no. 4: 226–239. 10.1038/s41579-023-00980-5. 37863969

[pei370182-bib-0052] Pliego, C. , F. Kamilova , and B. Lugtenberg . 2011. “Plant Growth‐Promoting Bacteria: Fundamentals and Exploitation.” In Bacteria in Agrobiology: Crop Ecosystems, 295–343. Springer. 10.1007/978-3-642-18357-7_11.

[pei370182-bib-0053] Rajkumar, M. , L. B. Bruno , and J. R. Banu . 2017. “Alleviation of Environmental Stress in Plants by Beneficial *Pseudomonas* spp.” Critical Reviews in Environmental Science and Technology 47, no. 6: 372–407. 10.1080/10643389.2017.1318619.

[pei370182-bib-0054] Ramesh, A. , S. K. Sharma , M. P. Sharma , N. Yadav , and O. P. Joshi . 2014. “Plant Growth‐Promoting Traits in *Enterobacter cloacae* Subsp. Dissolvens MDSR9 Isolated From Soybean Rhizosphere and Its Impact on Growth and Nutrition of Soybean and Wheat Upon Inoculation.” Agricultural Research 3, no. 1: 53–66. 10.1007/s40003-014-0100-3.

[pei370182-bib-0055] Ranadev, P. , A. Revanna , D. J. Bagyaraj , and A. H. Shinde . 2023. “Sulfur Oxidizing Bacteria in Agro Ecosystem and Its Role in Plant Productivity—A Review.” Journal of Applied Microbiology 134, no. 8: lxad161. 10.1093/jambio/lxad161. 37491695

[pei370182-bib-0056] Ríos‐Ruiz, W. F. , H. G. Jave‐Concepción , E. E. Torres‐Chávez , F. Rios‐Reategui , E. Padilla‐Santa‐Cruz , and N. E. Guevara‐Pinedo . 2025. “Plant‐Growth‐Promoting Microorganisms: Their Impact on Crop Quality and Yield, With a Focus on Rice.” International Journal of Plant Biology 16, no. 1: 9. 10.3390/ijpb16010009.

[pei370182-bib-0057] Rodríguez, H. , and R. Fraga . 1999. “Phosphate Solubilizing Bacteria and Their Role in Plant Growth Promotion.” Biotechnology Advances 17, no. 4–5: 319–339. 10.1016/S0734-9750(99)00014-2. 14538133

[pei370182-bib-0058] Rong, F. , Y. Lin , M. Zhang , Z. Cai , L. Wu , and F. Chen . 2025. “Straw‐Derived Bio‐Organic Fertilizer Enhanced Ecosystem Multifunctionality in Acidic Paddy by Improving Microbial Community Composition and Functional Diversity.” Journal of Environmental Management 390: 126314. 10.1016/j.jenvman.2025.126314. 40570423

[pei370182-bib-0059] Sandhya, V. , S. Z. Ali , M. Grover , G. Reddy , and B. Venkateswarlu . 2010. “Effect of PGPR on Compatible Solutes, Antioxidant Status and Growth of Maize Under Drought Stress.” Plant Growth Regulation 62: 21–30. 10.1007/s10725-010-9479-4.

[pei370182-bib-0060] Savci, S. 2012. “An Agricultural Pollutant: Chemical Fertilizer.” International Journal of Environmental Science and Development 3, no. 1: 73–76. 10.7763/IJESD.2012.V3.191.

[pei370182-bib-0061] Sharma, S. B. , R. Z. Sayyed , M. H. Trivedi , and T. A. Gobi . 2013. “Phosphate Solubilizing Microbes: Sustainable Approach for Managing Phosphorus Deficiency in Agricultural Soils.” Springerplus 2, no. 1: 587. 10.1186/2193-1801-2-587. 25674415 PMC4320215

[pei370182-bib-0062] Shen, X. , H. Hu , H. Peng , W. Wang , and X. Zhang . 2013. “Comparative Genomic Analysis of Four PGPR in *Pseudomonas* .” BMC Genomics 14: 271. 10.1186/1471-2164-14-271. 23607266 PMC3644233

[pei370182-bib-0063] Shultana, R. , A. T. K. Zuan , U. A. Naher , et al. 2022. “The PGPR Mechanisms of Salt Stress Adaptation and Plant Growth Promotion.” Agronomy 12, no. 10: 2266. 10.3390/agronomy12102266.

[pei370182-bib-0064] Singh, P. , R. K. Singh , Y. Zhou , et al. 2022. “Unlocking the Strength of Plant Growth Promoting Pseudomonas in Improving Crop Productivity in Normal and Challenging Environments: A Review.” Journal of Plant Interactions 17, no. 1: 220–238. 10.1080/17429145.2022.2029963.

[pei370182-bib-0065] Sneha, S. , B. Anitha , R. A. Sahair , et al. 2018. “Biofertilizer for Crop Production and Soil Fertility.” Academic Journal of Agricultural Research 6, no. 8: 299–306. 10.15413/ajar.2018.0130.

[pei370182-bib-0066] Spaepen, S. 2014. “Plant Hormones Produced by Microbes.” In Principles of Plant‐Microbe Interactions: Microbes for Sustainable Agriculture, 247–256. Springer International Publishing. 10.1007/978-3-319-08575-3_26.

[pei370182-bib-0069] Sultana, R. , S. M. N. Islam , and T. Sultana . 2023. “Arsenic and Heavy Metal Resistant Bacteria in Rice Ecosystem.” Environmental Technology & Innovation 31: 103160. 10.1016/j.eti.2023.103160.

[pei370182-bib-0068] Sultana, R. , S. M. N. Islam , N. Sriti , et al. 2024a. “ *Sphingomonas panaciterrae* PB20 Increases Growth, Photosynthetic Pigments, Antioxidants, and Mineral Nutrient Contents in Spinach ( *Spinacia oleracea* L.).” Heliyon 10, no. 3: e25596. 10.1016/j.heliyon.2024.e25596. 38356594 PMC10865318

[pei370182-bib-0067] Sultana, R. , S. M. N. Islam , S. B. Shuvo , et al. 2024b. “Endophytic Bacterium *Sphingomonas panaciterrae* NB5 Influences Soil Properties and Improves Growth, Nutrient Contents, and Yield of Red Amaranth ( *Amaranthus tricolor* L.).” Current Plant Biology 39: 100372. 10.1016/j.cpb.2024.100372.

[pei370182-bib-0070] Sultana, R. , A. I. I. Jashim , S. M. N. Islam , M. H. Rahman , and M. M. Haque . 2024c. “ *Pseudomonas mosselii* PR5 Improves Rice Growth and Yield.” BMC Plant Biology 24, no. 1: 1030. 10.1186/s12870-024-05649-6. 39478459 PMC11523849

[pei370182-bib-0071] Sun, J. , Q. Zhao , Y. N. Gao , et al. 2024. “Restoration of Degraded Seagrass Meadows: Effects of Plant Growth‐Promoting Rhizobacteria (PGPR) Inoculation on *Zostera marina* Growth, Rhizosphere Microbiome and Ecosystem Functionality.” Journal of Environmental Management 371: 123286. 10.1016/j.jenvman.2024.123286. 39531770

[pei370182-bib-0072] Tandon, H. L. S. , ed. 1993. Methods of Analysis of Soils, Plants, Waters and Fertilizers. FDCO.

[pei370182-bib-0073] Tanveer, S. , N. Akhtar , N. Ilyas , et al. 2023. “Interactive Effects of *Pseudomonas putida* and Salicylic Acid for Mitigating Drought Tolerance in Canola ( *Brassica napus* L.).” Heliyon 9, no. 3: e14193. 10.1016/j.heliyon.2023.e14193. 36950648 PMC10025117

[pei370182-bib-0074] Tarafder, S. , M. A. Rahman , M. A. Hossain , and M. A. H. Chowdhury . 2020. “Yield of *Vigna radiata* L. and Post‐Harvest Soil Fertility in Response to Integrated Nutrient Management.” Agricultural and Biological Sciences Journal 6, no. 1: 32–43.

[pei370182-bib-0075] Thammasittirong, S. N. R. , A. Thammasittirong , and S. Saechow . 2025. “Biocontrol and Growth Promotion of Rice by *Pseudomonas aeruginosa* SNTKU16: Beneficial Properties and Genomic Potential.” Journal of Microbiology and Biotechnology 35: e2411067. 10.4014/jmb.2411.11067. 39947704 PMC11876016

[pei370182-bib-0076] Tilman, D. , P. B. Reich , J. Knops , D. Wedin , T. Mielke , and C. Lehman . 2001. “Diversity and Productivity in a Long‐Term Grassland Experiment.” Science 294: 843–845. 10.1126/science.1060391. 11679667

[pei370182-bib-0077] Turino Mattos, M. L. , R. A. Valgas , and J. F. Martins . 2023. “Co‐Inoculation With Growth‐Promoting Bacteria Increases the Efficiency of Nitrogen Use by Irrigated Rice.” ACS Omega 8, no. 51: 48719–48727. 10.1021/acsomega.3c05339. 38162741 PMC10753571

[pei370182-bib-0078] Vyas, P. , and A. Gulati . 2009. “Organic Acid Production In Vitro and Plant Growth Promotion in Maize Under Controlled Environment by Phosphate‐Solubilizing Fluorescent Pseudomonas.” BMC Microbiology 9, no. 1: 174. 10.1186/1471-2180-9-174. 19698133 PMC2738680

[pei370182-bib-0079] Wang, M. , and Z. Xu . 2026. “PGPR‐Mediated Enhancement of Soil Nutrients, Rhizosphere Microbial Ecology, and Plant Growth: A Review.” Npj Biofilms and Microbiomes 12: 95. 10.1038/s41522-026-00966-0. 41866381 PMC13171949

[pei370182-bib-0080] Wu, B. , M. Zhang , Z. Zhai , et al. 2024. “Soil Organic Carbon, Carbon Fractions, and Microbial Community Under Various Organic Amendments.” Scientific Reports 14, no. 1: 25431. 10.1038/s41598-024-75771-w. 39455690 PMC11512027

[pei370182-bib-0081] Xiao, A. W. , Z. Li , W. C. Li , and Z. H. Ye . 2020. “Effect of PGPR on Arsenic Accumulation in Rice.” Chemosphere 242: 125136. 10.1016/j.chemosphere.2019.125136. 31654806

[pei370182-bib-0082] Yadava, U. L. 1986. “A Rapid and Nondestructive Method to Determine Chlorophyll in Intact Leaves.” HortScience 21, no. 6: 1449–1450. 10.21273/HORTSCI.21.6.1449.

[pei370182-bib-0083] Yasmin, S. , A. Zaka , A. Imran , et al. 2016. “PGPR‐Mediated Growth Promotion and Blight Suppression in Rice.” PLoS One 11, no. 8: e0160688. 10.1371/journal.pone.0160688. 27532545 PMC4988697

[pei370182-bib-0084] Zaidi, A. , M. Khan , M. Ahemad , and M. Oves . 2009. “Plant Growth Promotion by Phosphate Solubilizing Bacteria.” Acta Microbiologica et Immunologica Hungarica 56, no. 3: 263–284. 10.1556/amicr.56.2009.3.6. 19789141

[pei370182-bib-0085] Zhang, Q. , T. Guo , K. Sheng , et al. 2022. “Continuous Straw Return Mitigates Effects of Inorganic Fertilizers.” European Journal of Soil Science 73, no. 6: e13322. 10.1111/ejss.13322.

