# Supplementary material for: Application of Pseudomonas mosselii PR5 Enables Fertilizer Reduction in Rice While Maintaining Yield, Grain Quality, and Soil Nutrient Dynamics
Source: Plant Environ Interact. 2026 Jul 9;7(4):e70182. doi: 10.1002/pei3.70182 (PMC13346923; doi:10.1002/pei3.70182)
Supplement: Supplementary file 1 — Table S1: Physio‐chemical properties of initial soil. Table S2: Monthly average weather data for the experimental period (January–June 2024) obtained from the Bangladesh Agricultural University (BAU) Meteorological Station. Table S3: Summary of two‐way ANOVA results showing the effects of fertilizer level (F), bacterial inoculation (B), and their interaction (F × B) on plant growth, yield, grain nutritional quality, nutrient uptake, and soil physicochemical properties. F‐statistics and corresponding p values are presented for each variable. Figure S1: Effect of P. mosselii PR5 on (A) panicle length and (B) total grain weight of BRRI dhan29. Bars (mean ± standard error) with similar letter are not differed significantly according to Tukey's test (p < 0.05). B0 = no bacteria; B1 = seed priming (SP); B2 = seed priming + bacterial cell filtrate foliar application (SP + BCF) and F0 = 0% RDF (recommended dose of fertilizer); F1 = 50% RDF; F2 = 75% RDF; F3 = 100% RDF. [file PEI3-7-e70182-s001.docx]

**Application of *Pseudomonas mosselii* PR5 enables fertilizer reduction in rice while maintaining yield, grain quality, and soil nutrient dynamics**

**Supplementary Table and figure caption**

Table S1: Physio-chemical properties of initial soil.

Table S2: Monthly average weather data for the experimental period (January- June 2024) obtained from the Bangladesh Agricultural University (BAU) Meteorological Station.

Table S3**:** Summary of two-way ANOVA results showing the effects of fertilizer level (F), bacterial inoculation (B), and their interaction (F × B) on plant growth, yield, grain nutritional quality, nutrient uptake, and soil physicochemical properties. F-statistics and corresponding P values are presented for each variable.

Figure S1. Effect of *P. mosselii* PR5 on (A) panicle length and (B) total grain weight of BRRI dhan29. Bars (mean ± standard error) with similar letter are not differed significantly according to Tukey’s test (p <0.05). B0 = no bacteria; B1 = seed priming (SP); B2 = seed priming + bacterial cell filtrate foliar application (SP + BCF) and F0 = 0% RDF (recommended dose of fertilizer); F1 = 50% RDF; F2 = 75% RDF; F3 = 100% RDF.

**Table S1: Physio-chemical properties of initial soil**

| Parameter | Unit | Mean ± SE (n = 3) |
| --- | --- | --- |
| pH | - | 6.87 ± 0.158 |
| EC | µS/cm | 53.45 ± 2.49 |
| Sand | % | 20.33 ± 0.98 |
| Silt | % | 62 ± 0.94 |
| Clay | % | 17.67 ± 0.27 |
| Soil texture | Silt loam |  |
| Bulk density | g/cm^3^ | 1.3 ± 0.09 |
| Cation Exchange capacity | meq/100g | 10.13 ± 0.26 |
| Water holding capacity | % by volume | 40.5 ± 0.29 |
| Organic Carbon | % | 0.61 ± 0.05 |
| Organic Matter | % | 1.05 ± 0.08 |
| Total Nitrogen (N) | % | 0.05 ± 0.005 |
| Available Phosphorus (P) | µg/g | 5.29 ± 0.64 |
| Available Sulphur (S) | µg/g | 7.83 ± 0.42 |
| Exchangeable Potassium (K) | meq/100g | 0.2 ± 0.014 |
| Exchangeable Calcium (Ca) | meq/100g | 4.9 ± 0.53 |
| Exchangeable Magnesium (Mg) | meq/100g | 2.35 ± 0.24 |
| Available Iron (Fe) | µg/g | 18.27 ± 1.09 |
| Available Zinc (Zn) | µg/g | 1.60 ± 0.17 |

Table S2: Monthly average weather data for the experimental period (January- June 2024) obtained from the Bangladesh Agricultural University (BAU) Meteorological Station.

| Month | Air Temperature (°C) | | | Humidity (%) | | | Sunshine (hrs) |
| --- | --- | --- | --- | --- | --- | --- | --- |
|  | Max. | Min. | Mean | Max. | Min. | Mean |  |
| January | 21.74 | 13.00 | 17.4 | 97.22 | 65.19 | 86.54 | 2.99 |
| February | 26.62 | 15.73 | 21.20 | 95.31 | 47.75 | 76.44 | 5.93 |
| March | 29.81 | 18.74 | 24.29 | 94.38 | 51.45 | 76.67 | 6.94 |
| April | 35.08 | 24.78 | 29.95 | 93.93 | 51.46 | 76.5 | 7.19 |
| May | 33.97 | 24.79 | 29.47 | 92.74 | 56.80 | 77.32 | 6.39 |
| June | 31.68 | 25.83 | 28.78 | 95.76 | 74.76 | 86.5 | 2.80 |

Table S3: Summary of two-way ANOVA results showing the effects of fertilizer level (F), bacterial inoculation (B), and their interaction (F × B) on plant growth, yield, grain nutritional quality, nutrient uptake, and soil physicochemical properties. F-statistics and corresponding P values are presented for each variable.

**Agronomical Data:**

| **Parameters** | **Treatments** | **F value** | **P value** |
| --- | --- | --- | --- |
| Plant Height | Fertilizer levels (F) | 122.82 | 0.000 |
|  | Bacterial inoculation (B) | 33.17 | 0.000 |
|  | F x B | 1.67 | 0.172 |
| Total Tiller/Pot | F | 158.53 | 0.000 |
|  | B | 26.25 | 0.000 |
|  | F x B | 0.26 | 0.948 |
| Panicle Length | F | 23.94 | 0.000 |
|  | B | 7.88 | 0.002 |
|  | F x B | 2.57 | 0.046 |
| Root Length | F | 0.66 | 0.583 |
|  | B | 0.35 | 0.709 |
|  | F x B | 0.33 | 0.917 |
| Shoot Fresh Weight | F | 212.93 | 0.000 |
|  | B | 28.98 | 0.000 |
|  | F x B | 0.81 | 0.573 |
| Shoot Dry Weight | F | 128.92 | 0.000 |
|  | B | 27.24 | 0.000 |
|  | F x B | 3.06 | 0.023 |
| Root Fresh Weight | F | 59.32 | 0.000 |
|  | B | 22.23 | 0.000 |
|  | F x B | 1.02 | 0.435 |
| Root Dry Weight | F | 5201 | 0.000 |
|  | B | 21.99 | 0.000 |
|  | F x B | 1.3 | 0.295 |
| Panicle/Pot | F | 154.14 | 0.000 |
|  | B | 17.73 | 0.000 |
|  | F x B | 1.09 | 0.397 |
| Grain/Panicle | F | 11.19 | 0.000 |
|  | B | 23.69 | 0.000 |
|  | F x B | 0.96 | 0.000 |
| 100 Grain Weight | F | 12.04 | 0.000 |
|  | B | 4.1 | 0.029 |
|  | F x B | 14.31 | 0.000 |
| Total Grain/Pot | F | 208.46 | 0.000 |
|  | B | 22.17 | 0.000 |
|  | F x B | 0.86 | 0.535 |
| Total Grain Weight/Pot | F | 166.55 | 0.000 |
|  | B | 25.06 | 0.000 |
|  | F x B | 2.33 | 0.064 |
| Grain Yield/pot | F | 410.81 | 0.000 |
|  | B | 108.36 | 0.000 |
|  | F x B | 6.99 | 0.000 |
| Filled Grain/Pot | F | 117.61 | 0.000 |
|  | B | 32.1 | 0.000 |
|  | F x B | 1.07 | 0.407 |

| **Parameters** | **Treatments** | **F value** | **P value** |
| --- | --- | --- | --- |
| FL SPAD | Fertilizer levels (F) | 76.2 | 0.000 |
|  | Bacterial inoculation (B) | 10.32 | 0.000 |
|  | F x B | 1.76 | 0.151 |
| FL Chlorophyll *a* | F | 38.54 | 0.000 |
|  | B | 17.19 | 0.000 |
|  | F x B | 3.61 | 0.011 |
| FL Chlorophyll *b* | F | 88.44 | 0.000 |
|  | B | 69.91 | 0.000 |
|  | F x B | 2.89 | 0.029 |
| FL Chlorophyll total | F | 83.62 | 0.000 |
|  | B | 47.72 | 0.000 |
|  | F x B | 5.21 | 0.001 |

**Table S3. (Contd.)**

**Photosynthetic Pigments Data:**

**Proximate Data:**

| **Parameters** | **Treatments** | **F value** | **P value** |
| --- | --- | --- | --- |
| Protein | Fertilizer levels (F) | 144.62 | 0.000 |
|  | Bacterial inoculation (B) | 147.66 | 0.000 |
|  | F x B | 0.55 | 0.767 |
| Fat | F | 17.02 | 0.000 |
|  | B | 72.43 | 0.000 |
|  | F x B | 7.27 | 0.000 |
| Moisture | F | 4.07 | 0.018 |
|  | B | 32.86 | 0.000 |
|  | F x B | 4.03 | 0.006 |
| Ash | F | 10.09 | 0.000 |
|  | B | 16.01 | 0.000 |
|  | F x B | 3.44 | 0.014 |
| Carbs | F | 99.8 | 0.000 |
|  | B | 57.11 | 0.000 |
|  | F x B | 1.72 | 0.161 |

**Table S3. (Contd.)**

**Nutrients Data:**

| **Parameters** | **Treatments** | **F value** | **P value** |
| --- | --- | --- | --- |
| G_N | Fertilizer levels (F) | 12.75 | 0.000 |
|  | Bacterial inoculation (B) | 39.57 | 0.000 |
|  | F x B | 0.24 | 0.961 |
| Grain  P | F | 24.33 | 0.000 |
|  | PR5 | 7.9 | 0.002 |
|  | F x PR5 | 0.3 | 0.932 |
| Grain  K | F | 19.08 | 0.000 |
|  | PR5 | 4.76 | 0.018 |
|  | F x PR5 | 1.77 | 0.148 |
| Grain  S | F | 39.26 | 0.000 |
|  | PR5 | 36.68 | 0.000 |
|  | F x PR5 | 3.03 | 0.024 |
| Grain  Ca | F | 185.75 | 0.000 |
|  | PR5 | 112.74 | 0.000 |
|  | F x PR5 | 0.95 | 0.482 |
| Grain  Mg | F | 55.52 | 0.000 |
|  | PR5 | 99.42 | 0.000 |
|  | F x PR5 | 9.48 | 0.000 |
| Grain  Fe | F | 425.68 | 0.000 |
|  | PR5 | 1567.24 | 0.000 |
|  | F x PR5 | 111.22 | 0.000 |
| Grain  Zn | F | 37.32 | 0.000 |
|  | PR5 | 98.97 | 0.000 |
|  | F x PR5 | 13.51 | 0.000 |
| Grain  Mn | F | 20.97 | 0.000 |
|  | PR5 | 45.36 | 0.000 |
|  | F x PR5 | 1.65 | 0.177 |
| Flag Leaf  N | F | 60.53 | 0.000 |
|  | PR5 | 31.38 | 0.000 |
|  | F x PR5 | 3.51 | 0.012 |
| Flag Leaf  P | F | 39.91 | 0.000 |
|  | PR5 | 46.76 | 0.000 |
|  | F x PR5 | 2.08 | 0.094 |
| Flag Leaf  K | F | 66.93 | 0.000 |
|  | PR5 | 2.1 | 0.144 |
|  | F x PR5 | 3.23 | 0.018 |
| Flag Leaf  S | F | 27.8 | 0.000 |
|  | PR5 | 102.13 | 0.000 |
|  | F x PR5 | 0.86 | 0.540 |
| Flag Leaf  Ca | F | 25.41 | 0.000 |
|  | PR5 | 32.79 | 0.000 |
|  | F x PR5 | 1.4 | 0.254 |
| Flag Leaf  Mg | F | 11.6 | 0.000 |
|  | PR5 | 14.97 | 0.000 |
|  | F x PR5 | 0.17 | 0.982 |
| Flag Leaf  Fe | F | 283.7 | 0.000 |
|  | PR5 | 524.72 | 0.000 |
|  | F x PR5 | 20.53 | 0.000 |
| Flag Leaf  Zn | F | 338.38 | 0.000 |
|  | PR5 | 1763.05 | 0.000 |
|  | F x PR5 | 72.67 | 0.000 |
| Flag Leaf  Mn | F | 38.82 | 0.000 |
|  | PR5 | 196.21 | 0.000 |
|  | F x PR5 | 9.6 | 0.000 |
| Soil  N | F | 60.6 | 0.000 |
|  | PR5 | 83.6 | 0.000 |
|  | F x PR5 | 6.69 | 0.000 |
| Soil  P | F | 19.8 | 0.000 |
|  | PR5 | 128.72 | 0.000 |
|  | F x PR5 | 3.05 | 0.023 |
| Soil  K | F | 144.43 | 0.000 |
|  | PR5 | 44.25 | 0.000 |
|  | F x PR5 | 3.04 | 0.024 |
| Soil  S | F | 107.63 | 0.000 |
|  | PR5 | 49.54 | 0.000 |
|  | F x PR5 | 1.2 | 0.342 |
| Soil  Ca | F | 40.82 | 0.000 |
|  | PR5 | 90.97 | 0.000 |
|  | F x PR5 | 3.32 | 0.016 |
| Soil  Mg | F | 21.01 | 0.000 |
|  | PR5 | 23.27 | 0.000 |
|  | F x PR5 | 0.71 | 0.647 |
| Soil  Fe | F | 213.91 | 0.000 |
|  | PR5 | 225.08 | 0.000 |
|  | F x PR5 | 67.7 | 0.000 |
| Soil  Zn | F | 256.13 | 0.000 |
|  | PR5 | 114.74 | 0.000 |
|  | F x PR5 | 11.04 | 0.000 |
| Soil  Carbon | F | 12.04 | 0.000 |
|  | PR5 | 124.23 | 0.000 |
|  | F x PR5 | 1.32 | 0.000 |
| Soil  Organic Matter | F | 12.04 | 0.000 |
|  | PR5 | 124.23 | 0.000 |
|  | F x PR5 | 1.32 | 0.287 |

**Benefit Cost Ratio Data:**

| **Parameters** | **Treatments** | **F value** | **P value** |
| --- | --- | --- | --- |
| BCR | Fertilizer levels (F) | 332.85 | 0.000 |
|  | Bacterial inoculation (B) | 95.04 | 0.000 |
|  | F x B | 4.41 | 0.004 |

Note: F = fertilizer level; B = PR5 bacterial inoculation. Statistical significance was evaluated using two-way ANOVA, and treatment means were separated using Tukey’s HSD test at *p* ≤ 0.05.


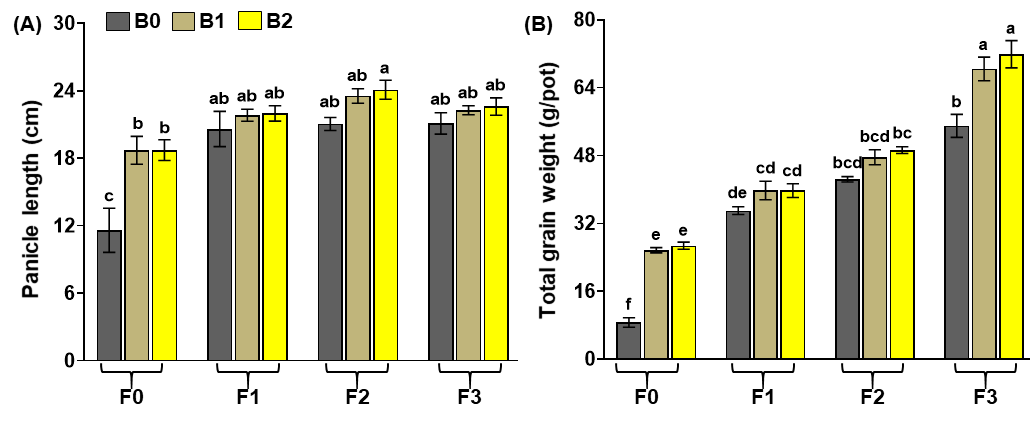
Figure S1. Effect of *P. mosselii* PR5 on (A) panicle length and (B) total grain weight of BRRI dhan29. Bars (mean ± standard error) with similar letter are not differed significantly according to Tukey’s test (p <0.05). B0 = no bacteria; B1 = seed priming (SP); B2 = seed priming + bacterial cell filtrate foliar application (SP + BCF) and F0 = 0% RDF (recommended dose of fertilizer); F1 = 50% RDF; F2 = 75% RDF; F3 = 100% RDF.
